# Epigenetic engineering reveals a balance between histone modifications and transcription in kinetochore maintenance

**DOI:** 10.1038/ncomms13334

**Published:** 2016-11-14

**Authors:** Oscar Molina, Giulia Vargiu, Maria Alba Abad, Alisa Zhiteneva, A. Arockia Jeyaprakash, Hiroshi Masumoto, Natalay Kouprina, Vladimir Larionov, William C. Earnshaw

**Affiliations:** 1 Wellcome Trust Centre for Cell Biology, University of Edinburgh, EH9 3QR, UK; 2 Department of Frontier Research, Laboratory of Cell Engineering, Kazusa DNA research Institute, Kisarazu 292-0818, Japan; 3 Genome Structure and Function Group, Developmental Therapeutics Branch, National Cancer Institute, National Institutes of Health, Bethesda, MD 20892, USA

## Abstract

Centromeres consist of specialized centrochromatin containing CENP-A nucleosomes intermingled with H3 nucleosomes carrying transcription-associated modifications. We have designed a novel synthetic biology ‘*in situ* epistasis' analysis in which H3 dimethylated on lysine 4 (H3K4me2) demethylase LSD2 plus synthetic modules with competing activities are simultaneously targeted to a synthetic alphoid^tetO^ HAC centromere. This allows us to uncouple transcription from histone modifications at the centromere. Here, we report that H3K4me2 loss decreases centromeric transcription, CENP-A assembly and stability and causes spreading of H3K9me3 across the HAC, ultimately inactivating the centromere. Surprisingly, CENP-28/Eaf6-induced transcription of the alphoid^tetO^ array associated with H4K12 acetylation does not rescue the phenotype, whereas p65-induced transcription associated with H3K9 acetylation does rescue. Thus mitotic transcription plus histone modifications including H3K9ac constitute the ‘epigenetic landscape' allowing CENP-A assembly and centrochromatin maintenance. H3K4me2 is required for the transcription and H3K9ac may form a barrier to prevent heterochromatin spreading and kinetochore inactivation at human centromeres.

## Abstract (web summary)

Centromeres are centrochromatin domains with CENP-A and H3 nucleosomes carrying transcription-associated modifications. Here the authors target synthetic modules to the centromeres to show that transcription plus histone modifications are required for CENP-A assembly and centrochromatin maintenance.

Centromeres are the genomic locus that directs chromosome segregation during cell division^1^. Human centromeres are characterized by the presence of extended arrays of α-satellite DNA, whose 171-bp monomers^2^ are organized into families of higher-order repeat (HOR) arrays in the core of the centromere^3^, where kinetochore assembly is nucleated. The conserved 17-bp CENP-B box sequence is distributed at regular positions within these HORs, and is the binding site for CENP-B (ref. 4). The centromeric HORs are flanked by divergent α-satellite monomers lacking CENP-B boxes and are rich in histone H3 trimethylated on lysine 9 (H3K9me3), which binds heterochromatin protein 1 (refs 5, 6, 7).

In Eukaryotes apart from Trypanosomatids^8^, regional centromeres^9^ are defined epigenetically by the presence of the centromere-specific histone H3 variant CENP-A^10^,^11^. Studies using stretched kinetochore chromatin fibres revealed that CENP-A-containing nucleosomes are localized to a subset of the α-satellite HOR repeats that ranges between 200 and 2,000 kb on different chromosomes and individuals^12^. In this centromeric ‘core' containing CENP-A, the canonical histone H3 bears modifications characteristic of actively transcribed regions, including dimethylation of lysine 4 (H3 dimethylated on lysine 4 (H3K4me2)) and lysine 36 (H3K36me2) (refs 13, 14, 15, 16). This so-called ‘centrochromatin'^14^ nucleates assembly of the kinetochore, a multi-protein complex that binds to microtubules and directs chromosome segregation^1^,^17^,^18^.

The presence of marks such as H3K4me2 or H3K36me2 places centrochromatin in the ‘yellow' chromatin class, which contains a broad range of active intergenic states^19^. Indeed, centromeric DNA has been shown to be transcribed, albeit at low levels^20^,^21^,^22^,^23^,^24^,^25^,^26^.

Our group previously constructed a synthetic human artificial chromosome (HAC) based on a dimeric α-satellite DNA array that contained alternating monomers with either CENP-B boxes or tetracycline operators (tetO)^27^,^28^,^29^. HACs are powerful tools for studying centromeres, as they are not essential for the life of the cell. The alphoid^tetO^ HAC centromere can be specifically engineered using chromatin modifiers fused to the tetracycline repressor (tetR). We have found that nucleating heterochromatin within centrochromatin disrupts kinetochore function^27^,^30^ and that low levels of transcription are needed to maintain an active kinetochore^16^,^31^.

In this work, we aim to study the role of centromeric transcription on CENP-A stability and kinetochore maintenance. To do this, we tether the H3K4-demethylase LSD2 to the alphoid^tetO^ HAC. LSD2 demethylates H3K4me2 in intragenic regions without recruiting other co-repressors^32^, as the best known H3K4 demethylase, LSD1 does^33^,^34^.

Importantly, we have exploited the multivalency of the alphoid^tetO^ HAC array to study chromatin requirements for CENP-A chromatin recruitment. We mapped dependencies using a novel ‘*in situ* epistasis' assay, in which pairs of chromatin-modifying activities are targeted simultaneously to the alphoid^tetO^ array. These assays allow us to uncouple transcription from histone-modification marks to study the role of centromeric transcription on kinetochore maintenance. Our results reveal that a balance of particular epigenetic modifications and transcriptional activity within centrochromatin regulate histone turnover and are essential for proper CENP-A incorporation and stability in human centromeres.

## Results

### LSD2 tethering to the alphoid^tetO^ HAC decreases H3K4me2

**Figure 1:**
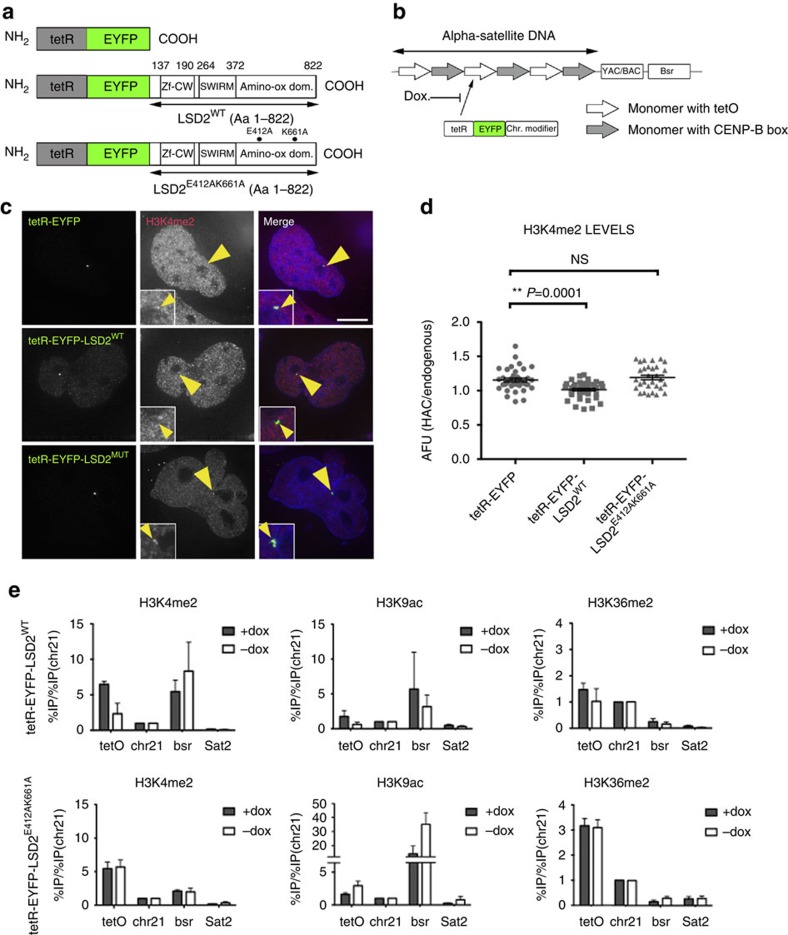
Tethering LSD2 to the alphoid^tetO^ HAC decreases the H3K4m2 levels. (**a**) Schematic drawings of the tetR-fusion constructs. (**b**) Schematic of the alphoid^tetO^ DNA array, derived from Nakano *et al.*^27^. (**c**) Immunofluorescence images of 1C7 cells expressing the indicated tetR-fusion proteins and staining for H3K4me2. Arrowheads depict the HAC as determined by the EYFP signal. Scale bar, 10 μm. (**d**) Quantification of fluorescence signals of HAC-associated H3K4me2 staining in individual cells transfected as in C plotted as arbitrary fluorescence units (AFU). Solid bars indicate the medians of three independent experiments and error bars represent the s.e.m. (**e**) ChIP analysis in 1C7 cells expressing tetR-EYFP-LSD2^WT^ (top) or tetR-EYFP-LSD2^E412AK661A^ (bottom) using the indicated antibodies. Data represents the levels of the indicated epigenetic marks in the presence of dox (grey bars) and after 3 days of dox washout (white bars). The alphoid^tetO^ HAC centromere (tetO), endogenous chromosome 21 centromere (chr21), the blasticidin resistance gene (bsr) and the degenerate satellite type-II (Sat2) repeats were assessed. Values were normalized to the chromosome 21 centromere and data represent the mean and s.d. of three independent experiments. Asterisks indicate a significant difference (*P*<0.05; Mann–Whitney's test).

To study the role of centromeric transcription in kinetochore maintenance, we removed the transcription-associated mark H3K4me2 from the alphoid^tetO^ HAC kinetochore. We did this by expressing a synthetic fusion construct encoding tetR-EYFP fused to lysine-specific histone demethylase 2 (tetR-EYFP-LSD2^WT^; Fig. 1a). A catalytically dead mutant of LSD2 fused to tetR-EYFP was also generated by introducing two mutations into the amino-oxidase domain (tetR-EYFP-LSD2^E412AK661A^; Fig. 1a; Supplementary Fig. 1a,b).

TetR-EYFP-LSD2^WT^ effectively removes H3K4me2 from the alphoid^tetO^ HAC (Fig. 1b). After transient expression of tetR-EYFP-LSD2^WT^ in 1C7 cells for 24 h immunofluorescence analysis detected significantly decreased levels of H3K4me2 on the alphoid^tetO^ HAC in cells and chromosome spreads (Fig. 1c,d and Supplementary Fig. 1c). In contrast, no significant differences in H3K4me2 levels were observed on the alphoid^tetO^ HAC in cells expressing either tetR-EYFP or tetR-EYFP-LSD2^E412AK661A^ (Fig. 1c,d). H3K4me2 staining was unaffected on all endogenous chromosomes after expressing any of these constructs. Thus, the LSD2 effects are specifically directed to the alphoid^tetO^ HAC centromere.

For further analyses, we generated 1C7 cell lines stably expressing either tetR-EYFP-LSD2^WT^ or tetR-EYFP-LSD2^E412AK661A^. Since H3K4me2 is associated with active chromatin regions^19^, we used chromatin immunoprecipitation (ChIP) followed by real-time quantitative PCR (RT-PCR) to analyse other marks typically associated with transcribed chromatin, including H3K9ac and H3K36me2. We compared the results obtained in the presence of doxycycline (no tethering) and after 3 days of doxycycline washout (tethering) for each mark.

Consistent with our immunofluorescence results, H3K4me2 levels fell after doxycycline washout in cells expressing tetR-EYFP-LSD2^WT^ but not in cells expressing the catalytically dead mutant tetR-EYFP-LSD2^E412AK661A^ (Fig. 1e). Levels of H3K9ac and H3K36me2 also fell in cells expressing tetR-EYFP-LSD2^WT^ (Fig. 1e, top), but not in cells expressing tetR-EYFP-LSD2^E412AK661A^ (Fig. 1e, bottom).

We conclude that tetR-EYFP-LSD2^WT^ specifically demethylates H3K4me2 at the alphoid^tetO^ HAC centromere without recruiting other factors, such as HDACs, which complicated earlier studies with LSD1 (ref. 16) due to CoREST binding^33^,^35^.

### Centromeric transcription decreases after H3K4me2 removal

**Figure 2:**
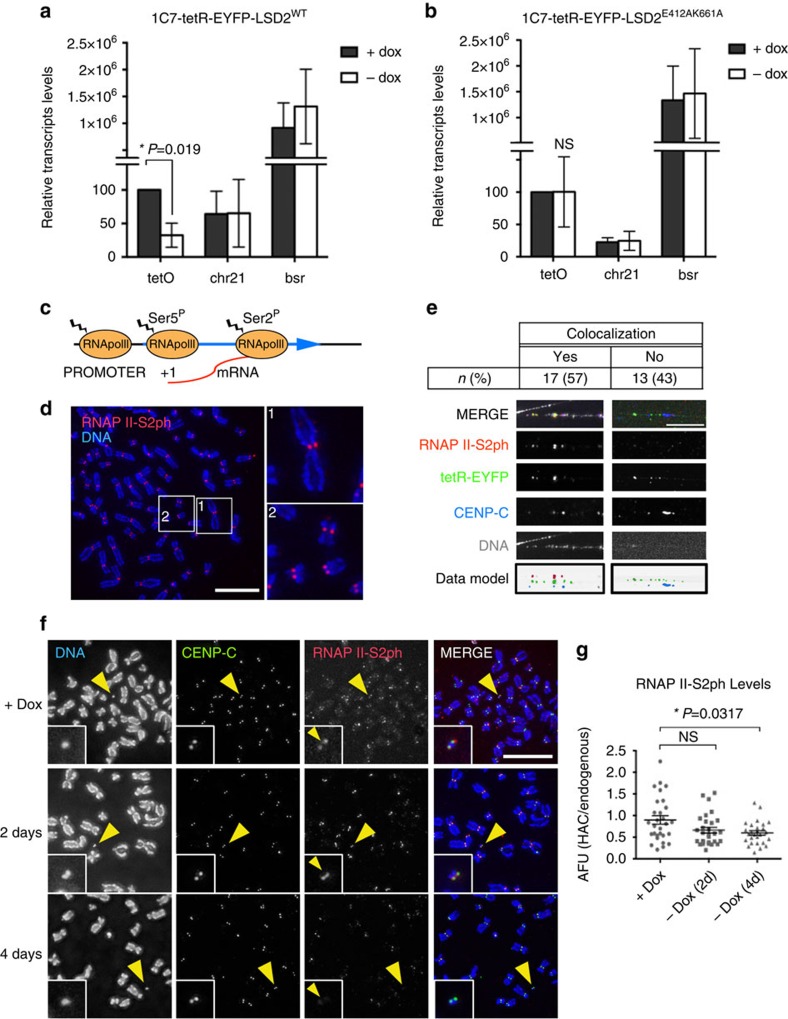
Tethering LSD2 to the alphoid^tetO^ HAC affects centromeric transcription. RT-PCR analysis of the centromeric transcripts in 1C7 cells expressing tetR-EYFP-LSD2^WT^ (**a**) or tetR-EYFP-LSD2^E412AK661A^ (**b**) in the presence of doxycycline (grey bars) and after 2 days of doxycycline washout (white bars). tetO (alphoid^tetO^ array), chr21 (centromere of chromosome 21) and bsr (Blasticidine resistance gene). Data represent the mean and s.e.m. of three independent experiments. (**c**) Schematic diagram representing the states of RNAP II during transcription. (**d**) Immunofluorescence analysis of unfixed 1C7 metaphase chromosomes stained with RNAP II-S2ph. DNA was counterstained with DAPI. Scale bar, 10μm (**e**) Analysis of RNAP II-S2ph with CENP-C on the HAC on stretched chromatin fibres. The HAC was detected with tetR-EYFP and DNA was counterstained with DAPI. Images show co-localization of RNAP II-S2ph and CENP-C on the HAC (left) and a fibre without RNAP II-S2ph signal (right). Bottom panels show a pseudocolored model using the inverted data from individual raw images. Scale bar, 5 μm. (**f**) Representative immunofluorescence images of 1C7 metaphase spreads expressing tetR-EYFP-LSD2^WT^ fusion protein in the presence of doxycycline (top) and after doxycycline washout at the indicated time points. Metaphase chromosomes were stained with DAPI, CENP-C and RNAP II-S2ph. Arrowheads depict the HAC. Scale bar, 10 μm. (**g**) Quantification of fluorescence signals of HAC kinetochore-associated RNAP II-S2ph staining in cells expressing tetR-EYFP-LSD2^WT^ in the presence of doxycycline and after doxycycline washout at the indicated time points. Values of the HAC kinetochore-associated RNAP II-S2ph signal were normalized for the mean of the RNAP II-S2ph signals at endogenous kinetochores. Solid bars indicate the medians and error bars represent the s.e.m. Results of two independent experiments were plotted together. Asterisks indicate a significant difference (*P*<0.05; Mann–Whitney's test).

Because H3K4me2 is associated with actively transcribed chromatin^19^, we analysed the levels of centromeric transcripts from the alphoid^tetO^ array by real-time RT-PCR using 1C7 cell lines stably expressing either tetR-EYFP-LSD2^WT^ or tetR-EYFP-LSD2^E412AK661A^ plus and minus doxycycline. Alphoid^tetO^ transcripts were significantly reduced after 2 days of doxycycline washout in cells expressing tetR-EYFP-LSD2^WT^ relative control cells (Fig. 2a, Supplementary Table 1). In contrast, no decrease in these transcripts was seen after tethering tetR-EYFP-LSD2^E412AK661A^ (Fig. 2b).

CENP-A occupies only a portion of the entire α-satellite array at centromeres^14^. To determine whether the alphoid^tetO^ transcription comes from within the CENP-A array or from flanking alphoid^tetO^ sequences, we examined the distribution of actively transcribing RNA polymerase II (phosphorylated at Serine 2 of the CTD: RNAP II-S2ph) relative to CENP-A (Fig. 2c).

Consistent with previous results from others^36^, we observed RNAP II-S2ph staining at centromeres in ∼50% of unfixed metaphase chromosome spreads (Fig. 2d). It is unclear why kinetochore-localized RNAP II-S2ph was consistently detected in only a subset of mitotic cells, although this could possibly be due to RNAP II stalling^37^,^38^. Immunofluorescence experiments on stretched chromatin fibres from mitotic 1C7 cells allowed us to map the distribution of RNAP II-S2ph and CENP-C across the kinetochore domain. Both signals co-localized on chromatin fibres, with the RNAP II-S2ph distribution slightly broader than the CENP-C domain (Supplementary Fig. 2a).

In controls, almost all (95%) chromatin fibres obtained after mitotic shake-off were positive for the mitotic marker H3S10ph (Supplementary Fig. 2b,c). Thus chromatin fibres obtained after mitotic shake-off do indeed come from mitotic chromosomes. Consistent with the presence of RNAP II-S2ph in metaphase chromosome spreads, we observed co-localization of RNAP II-S2ph and ACA signals in the 45% of chromatin fibres analysed (Supplementary Fig. 2d). Importantly, total ACA levels in individual fibres varied less than twofold, independent of the centromeric fibre length, suggesting that our fibre analysis is looking at single centromeres (Supplementary Fig. 2e).

Initial attempts to observe chromatin fibres derived from the alphoid^tetO^ HAC failed due to the loss of tetR binding during the procedure for stretching chromatin fibres. We overcame this problem by using purified tetR-EYFP fusion protein expressed in *E. coli* (Supplementary Fig. 2f) to stain the HAC *in vitro* after chromatin fibre stretching. *In vitro* staining with purified tetR-EYFP readily revealed the alphoid^tetO^ HAC in interphase and metaphase cells (Supplementary Fig. 2g,h). *In situ* tetR-EYFP staining using a different cell line containing an alphoid^tetO^ array integrated in a chromosome arm (HeLa 3-8_Int (ref. 39) confirmed the specificity of tetR-EYFP binding to tetO sequences (Supplementary Fig. 2i).

In stretched mitotic chromatin fibres, RNAP II-S2ph and CENP-C co-localized on the alphoid^tetO^ HAC (identified by tetR-EYFP binding) in 57% of the HAC fibres, consistent with the frequency of detection of RNAP II-S2ph signals in metaphase spreads (Fig. 2e and Supplementary Fig. 2j).

To test whether RNAP II-S2ph association with centromeres was affected by H3K4me2 removal, we repeated this analysis in 1C7 cells stably expressing tetR-EYFP-LSD2^WT^. Tethering tetR-EYFP-LSD2^WT^ to the alphoid^tetO^ HAC for 2 days caused a mild reduction in RNAP II-S2ph that became statistically significant 4 days after doxycycline washout (Fig. 2f,g).

We conclude that removal of H3K4me2 inhibits interphase and mitotic transcription at the alphoid^tetO^ HAC centromere.

### H3K4me2 removal disrupts the kinetochore at the HAC

**Figure 3:**
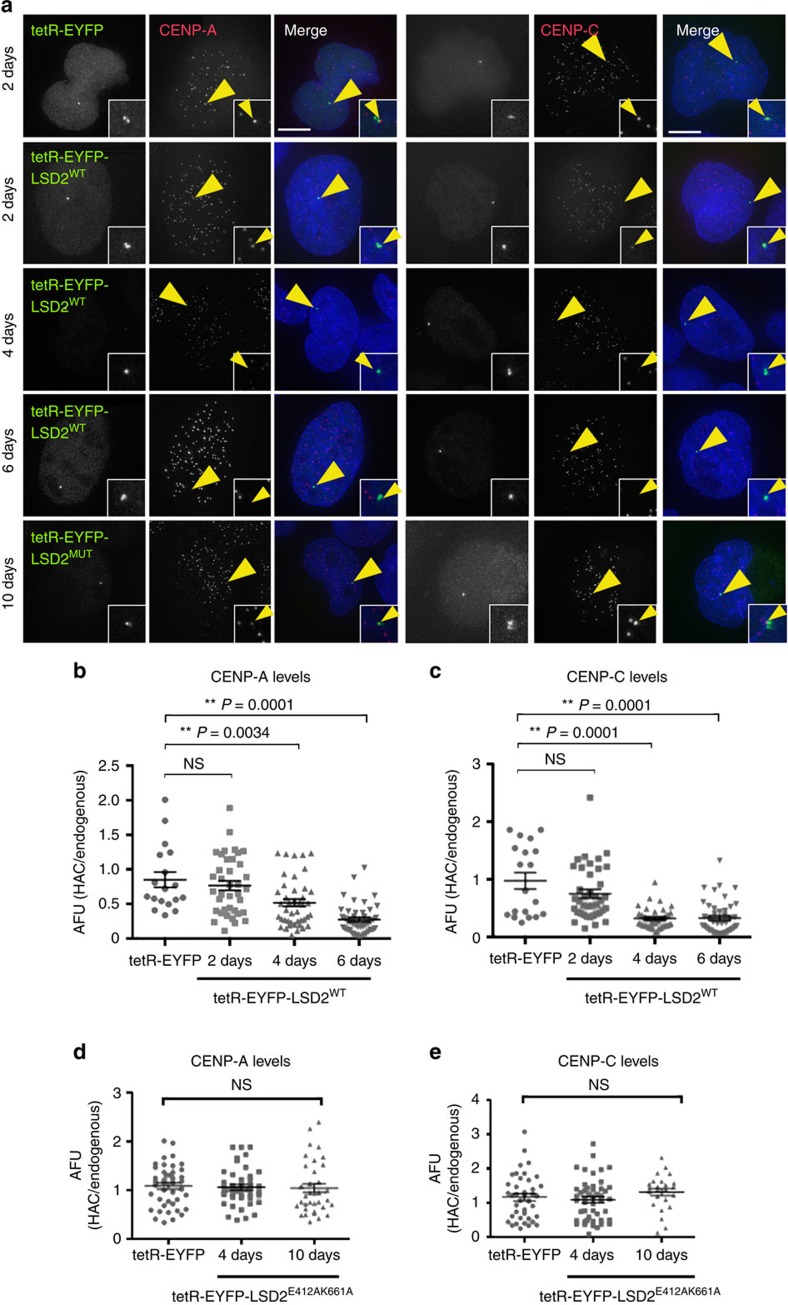
Tethering LSD2 to the alphoid^tetO^ HAC centromere affects the kinetochore assembly. (**a**) Immunofluorescence analysis of 1C7 cells expressing the indicated tetR-EYFP fusion proteins at the indicated time points and stained for either CENP-A (left) or CENP-C (right). Arrowheads depict the HAC. Scale bar, 10 μm. (**b**–**e**) Fluorescence signals of HAC-associated CENP-A (**b**,**d**) and CENP-C (**c**,**e**) staining in individual cells transfected with the indicated tetR-fusion constructs were quantified and plotted as AFU. Solid bars indicate the medians and error bars represent the s.e.m. *N*=two independent experiments for each time point and staining. Asterisks indicate a significant difference (*P<0.05; **P<0.01; Mann–Whitney's test).

Expression of tetR-EYFP-LSD2^WT^ for 2 days caused a slight decrease in CENP-A levels at the alphoid^tetO^ HAC centromere (Fig. 3a,b). In control cells expressing tetR-EYFP, the CENP-A signal on the alphoid^tetO^ HAC remained similar to that at endogenous centromeres (Fig. 3a). The drop in CENP-A levels in cells expressing tetR-EYFP-LSD2^WT^ became strongly significant after 4 days (Fig. 3a,b). In another control, binding of tetR-EYFP-LSD2^E412AK661A^ did not affect CENP-A levels on the alphoid^tetO^ HAC (Fig. 3a,d). Thus, long-term tetR tethering with catalytically dead LSD2 has no deleterious effect on the alphoid^tetO^ HAC kinetochore structure.

These observations of CENP-A were confirmed in parallel experiments staining for CENP-C. Expression of tetR-EYFP fusion protein had no effect on CENP-C levels at the alphoid^tetO^ HAC centromere (Fig. 3a). Tethering tetR-EYFP-LSD2^WT^ caused CENP-C levels to drop slightly after 2 days, and dropped markedly after 4 and 6 days (Fig. 3a,c). Control tethering of tetR-EYFP-LSD2^E412AK661A^ for up to 10 days had no significant effect on CENP-C levels (Fig. 3a,e).

The loss of CENP-A after H3K4me2 removal from the alphoid^tetO^ HAC centromere is due at least in part to defects in loading newly synthesized CENP-A. Levels of newly synthesized CENP-A-SNAP^40^ at the alphoid^tetO^ HAC centromere were significantly decreased after tethering tetR-EYFP-LSD2^WT^ relative to the tethering controls (Supplementary Fig. 3b,c) in pulse-chase experiments (Supplementary Fig. 3a). Expression of tetR-EYFP or tetR-EYFP-LSD2^E412AK661A^ did not affect CENP-A loading (Supplementary Fig. 3b,c). These data confirm our previous observation^16^, and suggest that H3K4me2 is required for loading newly synthesized CENP-A molecules at centromeres.

**Figure 4:**
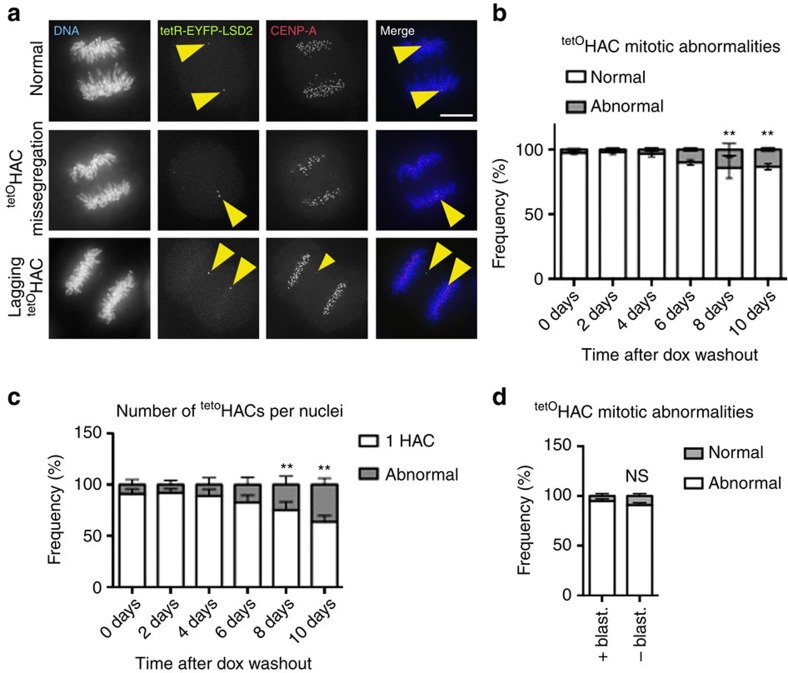
LSD2 activity at the alphoid^tetO^ HAC centromere affects kinetochore function and leads to chromosome segregation defects. (**a**) Representative immunofluorescence images of mitotic 1C7 cells expressing the tetR-EYFP-LSD2^WT^ fusion protein and stained for CENP-A. Images show examples of normal (top) and abnormal alphoid^tetO^ HAC segregation (middle and bottom rows). Arrowheads depict the HAC. Scale bar, 10 μm. (**b**) Analysis of the frequency of normal and abnormal alphoid^tetO^ HAC segregation at the indicated time points. Data represent the mean (and s.e.m.) of three independent assays of each time point after doxycycline washout (*n*=25 mitosis per time point and experiment; 0 days versus each time point, **P<0.0001; *χ*^2^-test). (**c**) Quantification of alphoid^tetO^ HAC copy-numbers as determined by the EYFP spot in interphase nuclei. Data represent the mean (and s.e.m.) of three independent assays of each time point after doxycycline washout (*n*=1,000 nuclei per time point and experiment; 0 days versus each time point, **P<0.0001; *χ*^2^-test). (**d**) Analysis of the frequency of normal and abnormal alphoid^tetO^ HAC segregation in the presence and absence of selection (Blasticidin) for 10 days. The alphoid^tetO^ HAC was identified by *in situ* tetR-EYFP tethering (see Supplementary Fig. 2). Data represent the mean (and s.e.m.) of three independent assays.

Analysis of the mitotic segregation of the alphoid^tetO^ HAC after tethering tetR-EYFP-LSD2^WT^ for up to 10 days confirmed that the decreased levels of CENP-A and CENP-C impair kinetochore function. We assessed HAC segregation during mitotic exit by tracking the EYFP signal on the alphoid^tetO^ array (Fig. 4a). The unperturbed alphoid^tetO^ HAC segregates accurately for up to 10 days after removal of blasticidin selection (Fig. 4d).

Tethering of tetR-EYFP-LSD2^WT^ causes a progressive increase in the frequency of alphoid^tetO^ HAC segregation abnormalities over time (Fig. 4b,c). Interestingly, although CENP-A and CENP-C levels fell significantly after 4 days of tethering, the frequency of alphoid^tetO^ HAC segregation errors became significant only at 8 days (Fig. 4b,c). This is consistent with reports that centromeres contain more CENP-A than is required for kinetochore assembly^41^,^42^.

We conclude that H3K4me2 is necessary for kinetochore assembly and function, probably due to its role in promoting centromeric transcription.

### Histone H4ac fails to maintain kinetochore without H3K4me2

Histone marks characteristic of ‘open' chromatin states, including acetylation, are consistent with kinetochore function^31^ and can increase the efficiency of *de novo* kinetochore formation^39^. To test the hypothesis that kinetochore defects caused by H3K4me2 removal could be prevented by acetylating the chromatin of the alphoid^tetO^ HAC centromere, we developed an ‘*in situ* epistasis' protocol in which we targeted two competing activities to the same alphoid^tetO^ DNA array. In a control for this approach, we found that simultaneous targeting of tetR-EYFP-LSD2^WT^ and the CENP-A chaperone tetR-mCherry-HJURP to the same alphoid^tetO^ DNA array rescued CENP-A targeting, while H3K4me2 levels remained low (Supplementary Fig. 4).

To determine whether H3K4me2 is epistatic over H4 acetylation for kinetochore stability, we simultaneously targeted tetR-EYFP-LSD2^WT^ and tetR-mCherry-CENP-28/Eaf6 to the HAC centromere. CENP-28 is a component of the HBO1 and MOZ/MORF histone acetyltransferase complexes^43^ and is required for efficient H4K12 acetylation on isolated mitotic chromosomes (I. Samejima and W.C.E., unpublished data).

Transient expression of tetR-mCherry-CENP-28/Eaf6 in 1C7 cells for 24 h significantly increased H4K12ac levels on the alphoid^tetO^ HAC compared with controls (Supplementary Fig. 5a,b). No changes were observed in the levels of H3K9 acetylation in these experiments (Supplementary Fig. 5d,e). Thus, tethering CENP-28/Eaf6 to the alphoid^tetO^ HAC selectively results in acetylation of histone H4K12.

**Figure 5:**
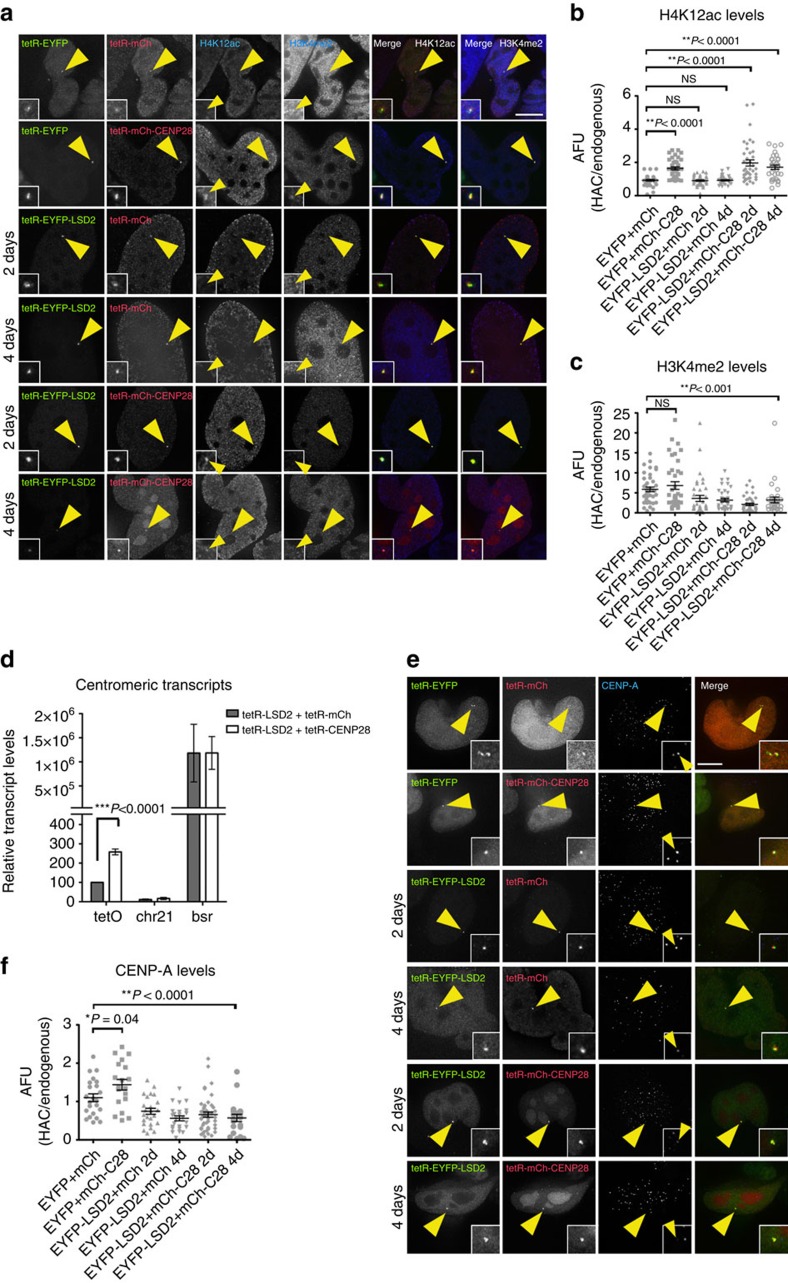
Tethering a transcriptional activator to the alphoid^tetO^ HAC centromere is not sufficient for kinetochore maintenance in the absence of H3K4me2. (**a**) Representative immunofluorescence images of 1C7 cells expressing the indicated tetR-fusion proteins at the indicated time points and staining for H4K12ac (panel 3) and H3K4me2 (panel 4). Merged images represent the overlay of EYFP and mCherry signals with H4K12ac (panel 5) or H3K4me2 (panel 6). Quantification of fluorescence signals of HAC-associated H4K12ac (**b**) and H3K4me2 (**c**) staining in individual cells transfected as in **a**. Solid bars indicate the medians and error bars represent the s.e.m. *N*=two independent experiments per time point and staining. (**d**) RT-PCR analysis of the centromeric transcripts in 1C7 cells expressing tetR-EYFP-LSD2^WT^ together with either tetR-mCherry (grey bars) or tetR-mCherry-CENP-28 (white bars). tetO (alphoid^tetO^ array), chr21 (centromere of chromosome 21) and *bsr* (Blasticidine resistance gene). Data represent the mean and s.e.m. of three independent experiments. (**e**) Representative immunofluorescence images of 1C7 cells expressing the indicated tetR-fusion proteins at the indicated time points and stained for CENP-A. (**f**) Quantification of HAC-associated CENP-A signals in individual cells transfected with the indicated tetR- fusion constructs. The values of the HAC-associated CENP-A signals were normalized for the mean of the CENP-A signals of endogenous centromeres. Solid bars indicate the medians and error bars represent the s.e.m. *N*=two independent experiments per time point and staining. Asterisks indicate significant differences (*P<0.05; **P<0.01; Mann–Whitney's test). Scale bars, 10 μm.

Co-tethering tetR-EYFP plus tetR-mCherry-CENP-28/Eaf6 increased CENP-A levels at the alphoid^tetO^ HAC centromere (Fig. 5e,f), consistent with previous observations that ‘open' chromatin favours CENP-A assembly^39^. This confirms that tetR-mCherry-CENP-28/Eaf6 is not detrimental to CENP-A assembly or maintenance. Levels of CENP-C at the alphoid^tetO^ HAC centromere were unaffected by this tethering (Supplementary Fig. 5f,g).

Co-expression of tetR-EYFP-LSD2^WT^ plus tetR-mCherry-CENP-28/Eaf6 in 1C7 cells for 2 and 4 days significantly increased H4K12ac levels on the alphoid^tetO^ HAC (Fig. 5a,b), despite H3K4me2 levels remaining low (Fig. 5a,c). Centromeric transcripts were significantly increased compared with levels observed after tethering the tetR-mCherry control (Fig. 5d; Supplementary Table 1). Thus, CENP-28/Eaf6 induces centromeric transcription even in the absence of H3K4me2. Measurement of EYFP and mCherry signals on the same alphoid^tetO^ HAC confirmed the equal binding of the chimeric proteins, tetR-EYFP and tetR-mCherry (Fig. 5a, Supplementary Fig. 5c).

Despite this rescue of centromeric transcription, we observed a highly significant drop in CENP-A and CENP-C levels on the alphoid^tetO^ HAC centromere at 2 and 4 days after transfection of tetR-EYFP-LSD2^WT^ plus tetR-mCherry-CENP-28/Eaf6 compared with control experiments (Fig. 5e,f; Supplementary Fig. 5f,g).

Thus, it is possible to target two modifiers to the same alphoid-DNA array and to observe their combinatorial effects. Furthermore, the loss of H3K4me2 from centromeric chromatin is epistatic over CENP-28/Eaf6-induced transcription and cannot simply be compensated by increasing centromere transcription or by acetylation of H4K12.

### Histone H3ac bypasses H3K4me2 requirement at kinetochore

Centromeric transcription during mitosis is necessary for kinetochore assembly and function^36^ and centromeric transcripts are essential for maintaining a functional kinetochore^26^,^38^,^44^,^45^,^46^,^47^. Here, we have shown that transcriptional activation coupled with histone H4 acetylation is not sufficient to maintain a functional kinetochore in the absence of H3K4me2. Thus, in addition to the process of transcription and/or the transcripts themselves, transcription-associated modifications of histone H3 might be essential for kinetochore maintenance. To test this hypothesis, we asked whether a transcriptional activator that increases H3K9 acetylation could stabilize the alphoid^tetO^ HAC kinetochore after H3K4me2 removal.

**Figure 6:**
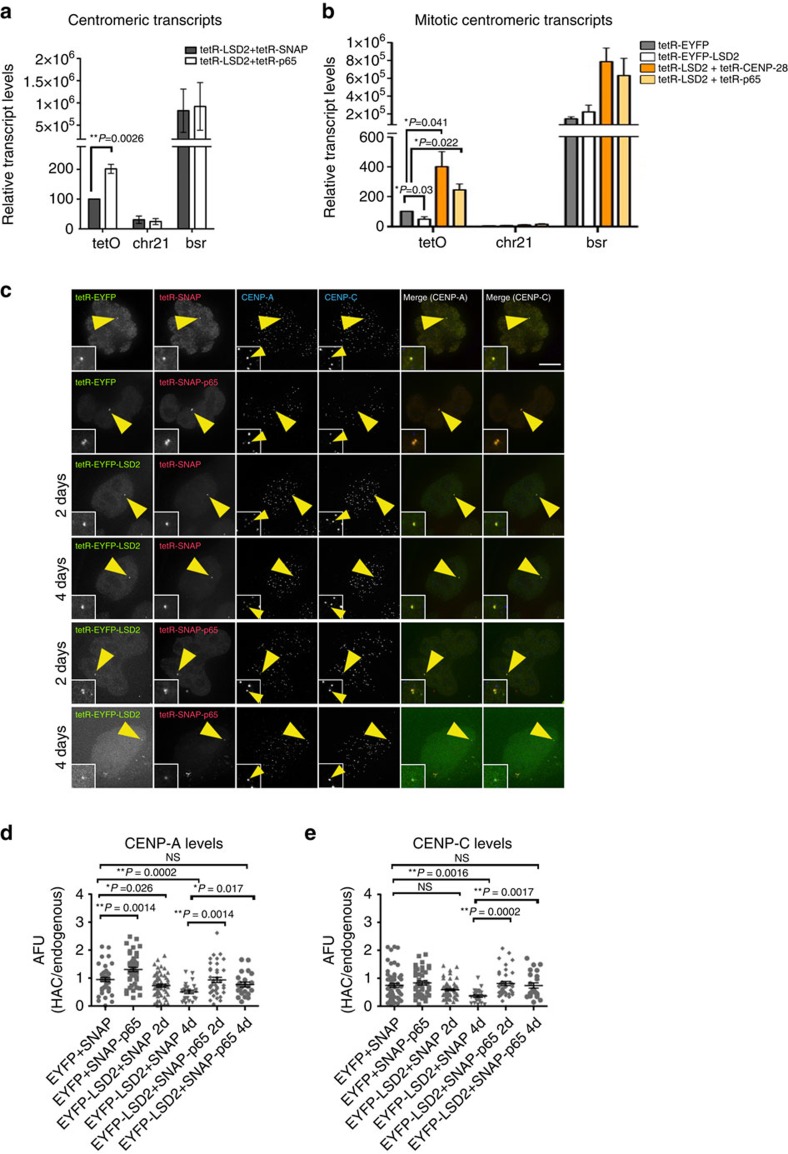
LSD2 effects on the alphoid^tetO^ HAC kinetochore are recovered by tethering a transcriptional activator that hyperacetylates histone H3K9. (**a**) RT-PCR analyses of 1C7 cells transfected with tetR-EYFP-LSD2^WT^ with either tetR-SNAP (grey bars) or tetR-SNAP-p65 (white bars). Expression levels of the alphoid^tetO^ array (tetO), chromosome 21 centromere (chr21) and blasticidin resistance gene (*bsr*) were normalized to those of β-actin. Data represent the mean and s.e.m. of three independent experiments. (**b**) RT-PCR analysis of centromeric transcripts in 1C7 cells after mitotic shake-off and expressing tetR-EYFP (grey bars), tetR-EYFP-LSD2^WT^ together with either tetR-mCherry (white bars), tetR-mCherry-CENP-28 (orange bars) or tetR-SNAP-p65 (yellow bars). tetO (alphoid^tetO^ array), chr21 (centromere of chromosome 21) and *bsr*. Data represent the mean and s.e.m. of three independent experiments. (**c**) Immunofluorescence images of 1C7 cells expressing the indicated tetR-fusion proteins at the indicated time points and staining for CENP-A and CENP-C. Merged images represent the overlay of EYFP and TMR-Star signals with CENP-A (panel 5) or CENP-C (panel 6). Quantification of fluorescence signals of HAC-associated CENP-A (**d**) and CENP-C (**e**) staining in individual cells transfected with the indicated tetR-fusion constructs. Values for the HAC-associated CENPs signals were normalized for the mean of the CENPs signals of endogenous centromeres. Solid bars indicate the medians and error bars represent the s.e.m. *N*=two independent experiments per time point and staining. Asterisks indicate significant differences (*P<0.05; **P<0.01; Mann–Whitney's test). Scale bar, 10 μm.

TetR-EYFP-p65 (C-terminal transactivator domain) increases H3K9ac and centromeric transcription levels on the alphoid^tetO^ HAC 10-fold without affecting kinetochore stability^31^. We therefore co-expressed tetR-EYFP-LSD2^WT^ with either tetR-SNAP or tetR-SNAP-p65 (fusions with mCherry were not functional) in 1C7 cells for 2 days and quantitated centromeric transcript levels on the alphoid^tetO^ HAC by RT-PCR. Tethering tetR-SNAP-p65 plus tetR-EYFP-LSD2^WT^ increased HAC centromeric transcript levels twofold relative to controls tethering tetR-EYFP-LSD2^WT^ plus tetR-SNAP (Fig. 6a, Supplementary Table 1). Thus, p65 stimulates transcription of the HAC centromere even without H3K4me2, albeit less strongly than when H3K4me2 is present^31^.

These experiments were performed with exponentially growing cultures (predominantly interphase), but it has recently been reported that centromeric transcription is differentially regulated during mitosis^38^. Analysis of centromeric transcript levels on the alphoid^tetO^ HAC in cells in the presence of colcemid confirmed that the HAC centromere is indeed transcribed during mitosis (Fig. 6b; Supplementary Table 2). Importantly, the transcripts behave similarly in mitotic and unsynchronized cultures (Fig. 6b, Supplementary Table 2).

H3K9ac levels were significantly increased on alphoid^tetO^ HAC centromeres in cells expressing tetR-SNAP-p65 (Supplementary Fig. 6b,c) consistent with changes in levels of centromeric transcripts (Fig. 6a,b). In contrast, H4K12ac levels were unchanged (Supplementary Fig. 6f,g). p65 tethering also significantly increased centromeric CENP-A, but not CENP-C, levels on the HAC (Fig. 6c–e). In controls, tetR-EYFP-LSD2^WT^ reduced H3K4me2 levels even in the presence of tetR-SNAP-p65 (Supplementary Fig. 6d,e) and tetR-SNAP (Fig. 6c–e). Furthermore, equal levels of both enzymes bound to the alphoid^tetO^ HAC (Supplementary Fig. 6a).

Co-expression of tetR-SNAP-p65 plus tetR-EYFP-LSD2^WT^ for 2 and 4 days, rescued both CENP-A and CENP-C levels despite the loss of H3K4me2 (Fig. 6c–e). We conclude that p65-induced transcription bypasses the requirement for H3K4me2 in kinetochore assembly, possibly because it induces both transcription and hyperacetylation of H3K9.

### H3K4me2 and H3K9ac link H3 turnover with CENP-A loading

We have shown that transcription linked with elevated H3K9ac is sufficient to maintain kinetochore function in the absence of H3K4me2 but that transcription linked with elevated H4K12ac is not. We hypothesized that these differing acetylation states might alter histone H3 dynamics at the alphoid^tetO^ HAC centromere.

**Figure 7:**
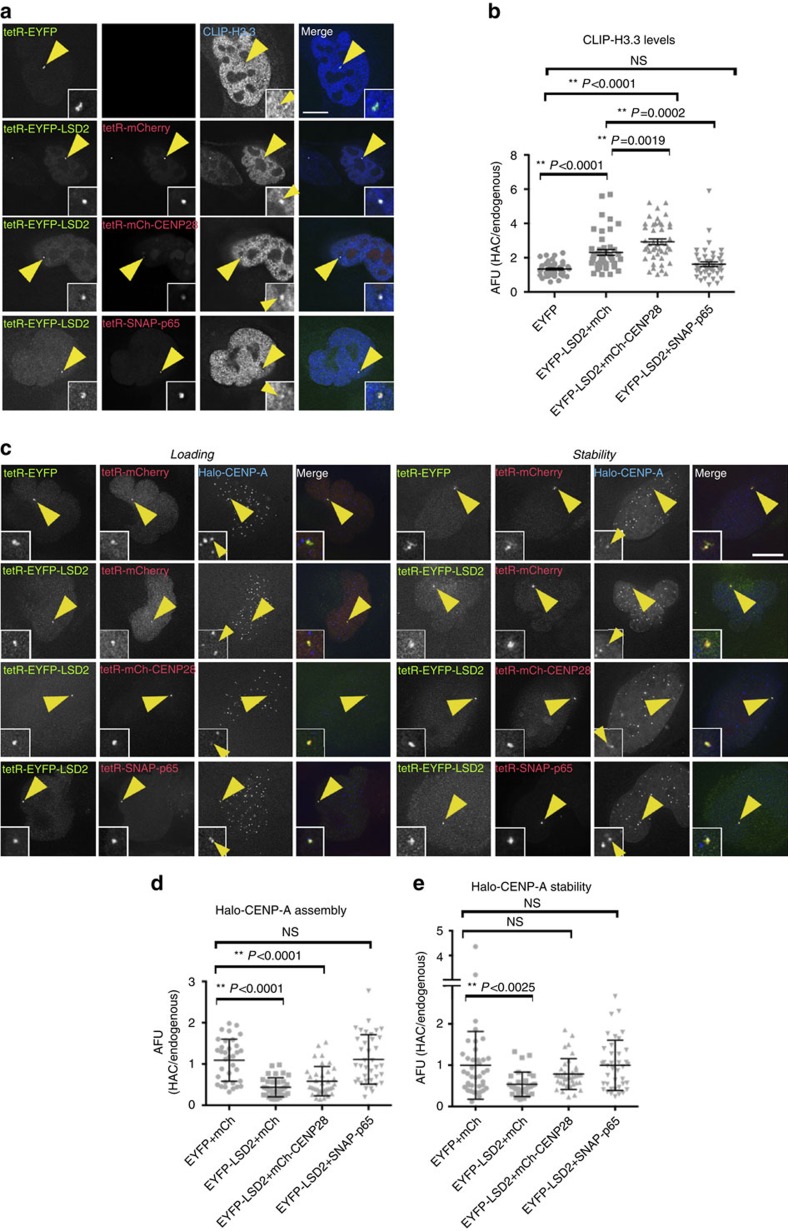
Histone H3 modifications stabilize centrochromatin for kinetochore maintenance. (**a**) Immunofluorescence images of 1C7 cells expressing CLIP-H3.3 and the indicated tetR-fusion proteins for 48 h. H3.3 was detected by staining for anti-CLIP (BC-Alexa647). Merged images represent the overlay of EYFP, mCherry/TMR-Star signals with Alexa647 (panel 4). (**b**) Quantification of fluorescence signals of HAC-associated CLIP-H3.3 staining in individual cells transfected with the indicated tetR-fusion constructs. Values for the HAC-associated CLIP-H3.3 signals were normalized for the mean of the H3.3 signals of the nuclei. (**c**) Representative images of 1C7 cells expressing Halo-CENP-A and the indicated tetR-fusion proteins for 48 h. Halo-CENP-A was detected with a Coumarin-tagged Halo ligand. Left panels show the loading of newly synthesized Halo-CENP-A and the right panels show levels of total Halo-CENP-A molecules at centromeres. (**d**,**e**) Quantification of Coumarin fluorescence signal associated with the HAC and normalized to the average signals at endogenous centromeres. Solid bars indicate the medians and error bars represent the s.e.m. *N*=three independent experiments. Scale bars, 10 μm.

We focused on histone H3.3, because its deposition is replication-independent and it was reported to be deposited at centromeres in S-phase as a placeholder for loading new CENP-A^48^. Indeed, we observed significantly increased levels of CLIP-H3.3 relative to controls on the HAC after tethering tetR-EYFP-LSD2^WT^ plus tetR-mCherry for 48 h (Fig. 7a,b). The most likely explanation for this result is that in the absence of H3K4me2, CENP-A incorporation is decreased and H3.3 placeholders remain.

Remarkably, transcription induced by p65 rescued the H3.3/CENP-A balance at centromeres in the absence of H3K4me2, whereas transcription of the same sequences induced by CENP-28/Eaf6 did not. Indeed, when tetR-EYFP-LSD2^WT^ was co-expressed with tetR-mCherry-CENP-28/Eaf6, CLIP-H3.3 levels were even higher than those observed with tetR-EYFP-LSD2^WT^ alone (Fig. 7a,b). In contrast, after tethering of tetR-EYFP-LSD2^WT^ plus tetR-SNAP-p65, CLIP-H3.3 returned to control levels on the alphoid^tetO^ HAC centromere (Fig. 7a,b).

We performed pulse-chase experiments expressing Halo-tagged CENP-A to distinguish whether the increased levels of histone H3.3 on the alphoid^tetO^ HAC centromere after H3K4me2 removal reflected a failure in CENP-A assembly or stability in centrochromatin. Halo-CENP-A loading was analysed using the protocol established for cells expressing CENP-A-SNAP (Supplementary Fig. 3a). The assay measuring Halo-CENP-A stability is described in the ‘Methods' section.

Consistent with previous results, both the incorporation and stability of Halo-CENP-A on the alphoid^tetO^ HAC centromere were significantly decreased after tethering tetR-EYFP-LSD2^WT^ plus tetR-mCherry but were rescued when tetR-EYFP-LSD2^WT^ was co-expressed together with tetR-SNAP-p65 (Fig. 7c–e).

A more complex picture emerged after expressing tetR-EYFP-LSD2^WT^ plus tetR-mCherry-CENP-28/Eaf6. This combination failed to rescue the incorporation of newly synthesized Halo-CENP-A (Fig. 7c,d). However, Halo-CENP-A stability was partly rescued—levels of Halo-CENP-A were no longer significantly different from control levels (Fig. 7c,e).

**Figure 8:**
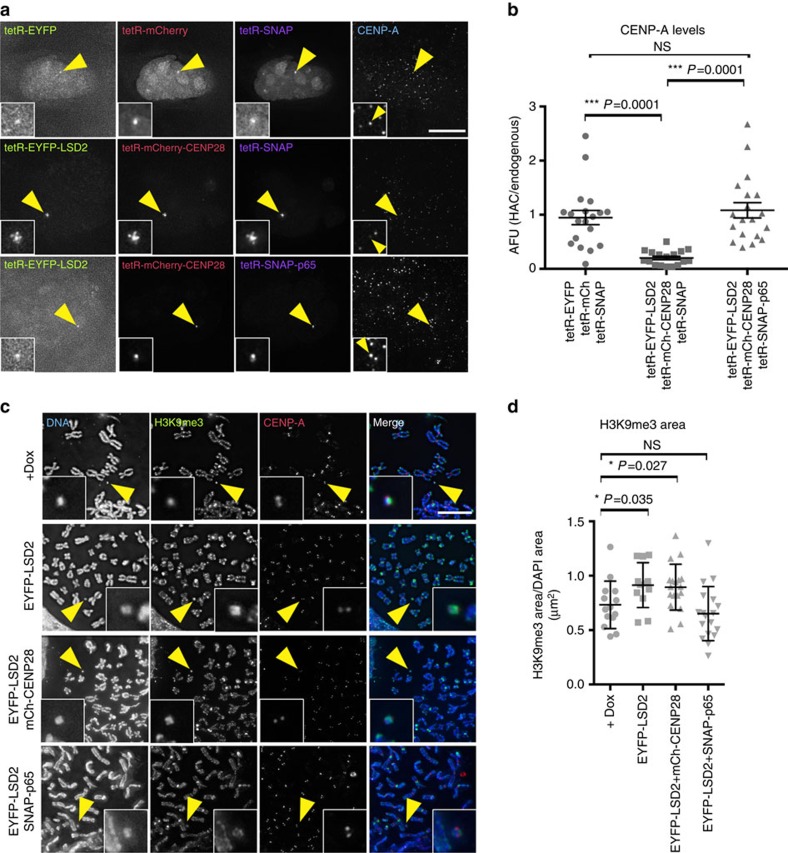
H3K4me2 and H3K9ac maintain the epigenetic signature of centrochromatin. (**a**) Representative images of 1C7 cells expressing the indicated tetR-fusion proteins for 48 h. The tetR-SNAP fusion proteins were detected by incubating cells with SNAP-Cell 647-SiR substrate (panel 3) and CENP-A was detected with Alexa-405-coupled antibodies (panel 4). (**b**) Quantification of fluorescence signals of HAC-associated CENP-A staining in individual cells transfected with the indicated tetR-fusion constructs. Values for the HAC-associated CENP-A were normalized for the mean of CENP-A signals on endogenous chromosomes. (**c**) Representative images of 1C7 metaphase spread expressing the indicated tetR-fusion proteins for 4 days. Metaphase chromosomes were stained with DAPI, CENP-A and H3K9me3. Arrowheads depict the HAC. (**d**) Quantification of the area occupied for H3K9me3 in the HAC normalized for the DAPI area in the presence of doxycycline (no tetR-EYFP binding) and after 4 days of expression of the indicated tetR-fusion proteins. Asterisks indicate significant differences (*P<0.05; **P<0.01; Mann–Whitney's test). Solid bars indicate the medians and error bars represent the s.e.m. *N*=two independent experiments. Scale bars, 10 μm.

Since both tetR-SNAP-p65 and tetR-mCherry-CENP-28/Eaf6 cause a similar increase in HAC centromere transcription, these results suggest that centromere transcription on its own is not sufficient to support CENP-A incorporation in the absence of H3K4me2. Alternatively, it could be suggested that tetR-mCherry-CENP-28/Eaf6 somehow actively destabilizes the centromere—perhaps by raising the level of centromeric transcription too high. Although this is unlikely, since tethering tetR-mCherry-CENP-28/Eaf6 on its own causes a significant increase in CENP-A levels (Fig. 5f), we tested this hypothesis by performing a three-way *in situ* epistasis experiment. Such three-way tethering is possible—the three fluorescent signals for EYFP, mCherry and 647-Sir could be observed on the same HAC (Fig. 8a).

CENP-A levels on the HAC centromere were fully restored when tetR-EYFP-LSD2^WT^ plus tetR-mCherry-CENP-28/Eaf6 were co-expressed together with tetR-SNAP-p65 for 2 days, but not after three-way tethering of tetR-EYFP-LSD2^WT^ plus tetR-mCherry-CENP-28/Eaf6 plus tetR-SNAP (Fig. 8a,b). These results strongly argue that the failure of tetR-mCherry-CENP-28/Eaf6 to rescue CENP-A incorporation is not due to a deleterious effect of the chimeric tetR-mCherry-CENP-28/Eaf6.

Since both tetR-mCherry-CENP-28/Eaf6 and tetR-SNAP-p65 rescue transcription of the centromere following loss of H3K4me2, but only tetR-SNAP-p65 fully rescues the assembly of new CENP-A at centromeres, the most likely explanation is that transcription associated with H3K9ac is required for centromere maintenance (at least in the absence of H3K4me2). However, it is also possible that other chromatin marks also contribute, since tetR-SNAP-p65, but not tetR-mCherry-CENP-28/Eaf6, rescues H3K36me2 levels, which are also decreased after H3K4me2 removal (Supplementary Fig. 7).

These results suggest that H3K4me2 and H3K9ac plus either transcription or possibly the centromeric transcripts themselves are important for the correct turnover of H3.3/CENP-A molecules and proper CENP-A loading.

### H3K4me2 prevents H3K9me3 spreading into centrochromatin

Given the correlation between H3K9 acetylation and centromere stability, we next asked whether H3K4me2 stabilizes the centromere by preventing heterochromatin spreading into centrochromatin. Normally, the CENP-A domain of endogenous chromosomes lacks detectable H3K9me3. Indeed, in the presence of doxycycline the CENP-A domain of the HAC in stable cell lines expressing tetR-EYFP-LSD2^WT^ lacked H3K9me3 staining, and comprised 27% of the total HAC area (Fig. 8c,d). In contrast, after doxycycline washout and tethering tetR-EYFP-LSD2^WT^ to the HAC for 4 days (a time point when kinetochore defects appear), H3K9me3 occupied an increased area of the HAC compared with controls (91 versus 73%; Fig. 8c,d). Furthermore, H3K9me3 began to spread into the area occupied by CENP-A.

This incursion of H3K9me3 into the centromere was rescued in cells simultaneously expressing tetR-EYFP-LSD2^WT^ plus tetR-SNAP-p65. In those cells, the area on the HAC occupied by H3K9me3 was even less than in controls and left the CENP-A domain devoid of H3K9me3 (65 versus 73%; Fig. 8c,d). In contrast, expression of tetR-EYFP-LSD2^WT^ plus tetR-mCherry-CENP-28/Eaf6 failed to prevent heterochromatin spreading into the kinetochore domain, as evidenced by the area occupied by H3K9me3 (86 versus 73% in the control; Fig. 8c,d).

Altogether, these results suggest that H3K4me2 plus transcription associated with H3K9ac antagonize heterochromatin spreading into centrochromatin.

## Discussion

Centromeres were long assumed to be composed of heterochromatin. However, a landmark study by Sullivan and Karpen^14^ showed that centromeric chromatin is characterized by the presence of CENP-A plus H3K4me2, a mark associated with RNAP II transcription. Subsequently, we showed that heterochromatin actually inactivates kinetochores^27^ and identified H3K36me2, a second transcription-linked mark, at centromeres^16^. Here, we used a synthetic HAC^27^ to examine the functional interplay between centromeric transcription, H3K4me2 and acetylation of histones H3 and H4 in centrochromatin maintenance. Our results suggest that centromeric transcription promoted by H3K4me2 is associated with H3K9 acetylation, and that this prevents spreading of H3K9me3 into the centromere.

**Figure 9:**
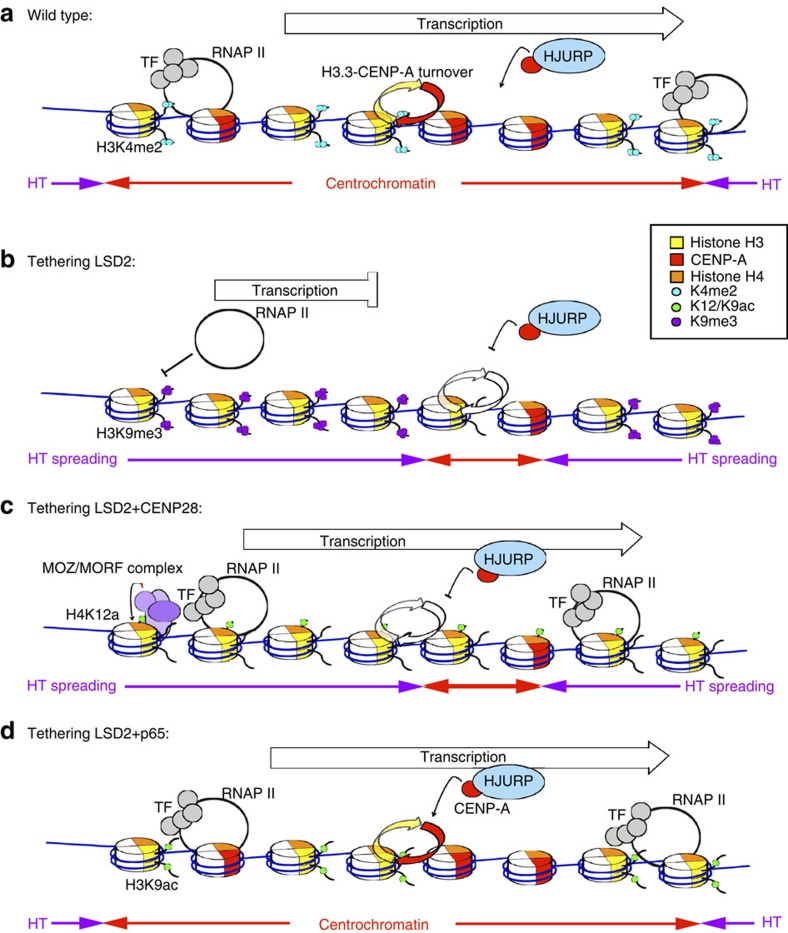
Model to explain the role of transcription at centrochromatin on kinetochore maintenance. CENP-A is represented in red, histone H3 is represented in yellow and histone H4 in orange. See text for details. (**a**) Model for centrochromatin maintenance in a normal (wild type) situation. (**b**–**d**) Model of the effects observed after engineering the alphoid^tetO^ HAC centromere. HT, heterochromatin; TF, transcription factors.

Recent results have revealed that centromeres undergo low levels of RNAP II-mediated transcription during mitosis^36^,^38^. We confirmed these results for the HAC and further showed that H3K4me2 depletion affects levels of both centromeric mitotic transcripts and centromere-associated RNAP II (Fig. 9a). Many transcription factors appear to read the H3K4 methylation mark: in one analysis, over 90% of transcription factor-binding sites were found to map within regions of increased H3K4 methylation^49^. Specifically, Sgf29 binding to H3K4me2/3 has been reported to recruit the SAGA complex and promote histone H3 acetylation^50^. At centromeres this acetylation could be linked with licensing for new CENP-A assembly, as seen when p300 and PCAF acetyltransferase domains were targeted to the alphoid^tetO^ array^39^. In addition, the chromatin remodeller CHD1 also binds H3K4me2 (ref. 51) and this could promote RNAP II activity associated with H3 acetylation at centromeres during mitosis. Indeed, CHD1 depletion has been shown to decrease CENP-A incorporation and disrupt centromere function^52^.

Centromeric transcription defects resulted in a progressive loss of CENP-A (Fig. 9b). These changes in CENP-A levels, although being statistically significant, were moderate. CENP-A is extremely stable at centromeres^53^, thus making it hard to observe large effects. Our detailed proteomic analysis of isolated mitotic chromosomes showed only moderate differences in CENP-A levels even when critical assembly factors such as HJURP or Mis18α were depleted for several days^54^. Kinetochores depleted of H3K4me2 remained functional for several days until CENP-A levels fell by >50%. This is consistent with reports that human centromeres contain a 2.5-fold excess of CENP-A^42^ and supports an emerging view that loss of centromeric transcription disrupts kinetochore assembly and leads to chromosome missegregation^26^,^45^,^46^,^55^. Importantly, decreased levels of CENP-A were always accompanied by decreased levels of CENP-C in independent experiments.

The linking of centromere stability to multiple chromatin marks and to the process of transcription (or to the transcripts themselves) reveals a complex system for centromere maintenance. Epigenetic marks may maintain centrochromatin stability by recruiting factors such as RSF^56^ and/or MgcRacGAP^57^. In other experiments, our laboratories recently found that the chromatin remodelling factor RSF, recruited by acetylation of histone H3, can promote CENP-A incorporation at an ectopic site^58^,^59^.

Given this complexity, it is a significant challenge to establish functional relationships between the multiple factors and processes involved in kinetochore maintenance. Here, we approached this problem by establishing novel ‘*in situ* epistasis' assays in which two or three competing activities were targeted to the same centromere, and the functional outcome determined. These assays allowed us to uncouple transcription from histone modifications present at the alphoid^tetO^ HAC centromere. All assays used LSD2 to lower H3K4me2 levels, coupled with activities that promote transcription associated with H4K12ac or with H3K9ac. Importantly, controls showed that individual synthetic modules all functioned as expected when targeted in combinations to the tetO array on the HAC. This approach increases the versatility of the tetO array approach to the analysis of chromatin states.

Previous studies suggested that H4K12ac might confer heterochromatin plasticity required for DNA repair and replication at pericentromeric and telomeric regions^60^,^61^. H3K14ac has also been reported to recruit RSF1 to centromeres^58^ and RSF has been reported to stabilize CENP-A incorporation in centrochromatin^56^. We found that tethering of CENP-28/Eaf6, which promotes HBO1 and MOZ/MORF-dependent acetylation of H4K12, resulted in increased transcription of the HAC centromere. This was associated with increased CENP-A incorporation at unperturbed centromeres containing H3K4me2, indicating that the levels of transcription induced by tethering CENP-28/Eaf6 are not incompatible with kinetochore maintenance. Importantly, this CENP-28/Eaf6-induced centromeric transcription failed to bypass the requirement for H3K4me2 in kinetochore maintenance (Fig. 9c). Thus, mitotic transcription alone is not the ultimate epigenetic signal that recruits CENP-A.

In contrast, p65-induced transcription, which was associated with acetylation of H3K9 but not H4K12, did bypass the requirement for H3K4me2 in kinetochore maintenance (Fig. 9d). This suggested that the balance of transcription-associated histone modifications might create an environment permissible for kinetochore maintenance. Indeed, tethering of p65 also restored H3K36me2 levels in the absence of H3K4me2.

CENP-A chromatin propagation is a multistep complex pathway involving chromatin licensing, loading of new CENP-A molecules and CENP-A stabilization at centromeres^62^. Our pulse-chase experiments expressing Halo-CENP-A in *in situ* epistasis assays allowed us to demonstrate that loss of H3K4me2 affected the loading and stability of CENP-A, and both could be rescued by p65-induced transcription. Importantly, while CENP-28/Eaf6-induced transcription failed to restore CENP-A loading on the HAC, it was able to stabilize CENP-A nucleosomes. Indeed, other authors showed that centromeric ncRNAs bind chromatin containing CENP-A^26^,^46^ and CENP-C^63^,^64^. It is possible that both the process of transcription and the centromeric transcripts themselves might be necessary for the regulation of chromatin remodelling, CENP-A assembly and centrochromatin maintenance.

Loss of H3K4me2 appeared to decrease the rate of histone H3.3 replacement by newly synthesized CENP-A molecules. Strikingly, the decreased rates of H3 replacement observed in the absence of H3K4me2 or H3K9ac were coupled with an increased level of H3K9me3 in the alphoid^tetO^ HAC. Transcription stimulated by p65 restored the normal levels of H3.3 replacement, and also the normal distribution of H3K9me3 on the HAC, decreasing H3K9me3 specifically on the kinetochore. This is consistent with H3K9ac acting as a barrier for heterochromatin spreading into centrochromatin.

The simplest interpretation of our results is that H3K4me2 facilitates transcription of centrochromatin that is linked to histone H3 acetylation (Fig. 9). Transcription of the alphoid^tetO^ array linked to acetylation of H4K12 is not sufficient to rescue CENP-A dynamics. This suggests that H3 acetylation either on its own or in combination with other factors, has at least one critical function in CENP-A assembly and centrochromatin maintenance. It may be part of a chromatin-targeting motif for the Mis18 complex to recruit HJURP and promote CENP-A insertion (thus maintaining the H3.3/CENP-A ratio). Alternatively, it may directly antagonize heterochromatin spreading, since H3K9ac can block the formation of H3K9me3.

In conclusion, our results suggest that a balance of mitotic transcription (including a possible role for the transcripts themselves), epigenetic modifications and chromatin remodelling in centrochromatin act as a barrier to prevent heterochromatin spreading and kinetochore inactivation in human centromeres.

## Methods

Expression constructs, detailed description of ChIP protocols and analysis are provided in Supplementary data.

### Cell culture and transfection

1C7 cells, a fusion of an HT1080-derivative cell line (ATCC CCL121) carrying the alphoid^tetO^ HAC (AB2.2.18.21) and HeLa cells (ATCC CCL-2)^30^ were maintained in DMEM medium supplemented with 5% fetal bovine serum (Invitrogen) and 100 U ml^−1^ penicillin G and 100 μg ml^−1^ streptomycin sulfate (Invitrogen). Blasticidin S (Invitrogen) was added to a final concentration of 4 μg ml^−1^ to maintain the alphoid^tetO^ HAC. Cells were grown at 37 °C in 5% CO_2_ in a humidified atmosphere.

Transfections were performed using Xtremegene-9 (Roche) following manufacturer's instructions. In brief, for transfections of cells growing in 12-well plates, transfection complexes containing 3 μl Xtremegene-9 reagent and 1 μg plasmid DNA were prepared in 100 μl OptiMEM (Invitrogen). After 20 min of incubation at room temperature (RT), 50 μl of transfection complexes were added drop-wise in each well. For transient expression experiments, transfectant cells were selected by incubating cells with 2 μg ml^−1^ of Puromycin (Sigma) for 24 h.

To generate 1C7 stable cell lines expressing tetR-EYFP-LSD2^WT^ and tetR-EYFP-LSD2^E412AK661A^, cells were transfected with the tYIP-LSD2^WT^ and tYIP-LSD2^E412AK661A^ constructs. Transfected cells were selected adding 2 μg ml^−1^ of Puromycin (Sigma), 4 μg ml^−1^ Blasticidin S and 1 μg ml^−1^ doxycycline (Sigma). Clonal cell lines were isolated by limiting dilution in 96-well plates and grown in the same selective media. Nuclear localization and targeting to the alphoid^tetO^ HAC was confirmed by fluorescence microscopy. Doxycycline washout time course experiments were started with a subconfluent 1C7 culture stably expressing either tYIP-LSD2^WT^ or tYIP-LSD2^E412AK661A^ constructs and grown in the presence of the above drugs. Cells were washed three times with warm D-PBS (invitrogen), followed by incubation in drug-free DMEM for 30 min at 37 °C. Next, cells were washed three times with D-PBS before drug-free DMEM was added allowing tetR-fusion protein binding to the alphoid^tetO^ array.

### Immunostaining and cytological analysis

Indirect immunofluorescence staining of cells fixed in 2.6% Formaldehide/1 × phosphate-buffered saline (PBS) was performed following standard procedures. The following antibodies were used: rabbit anti-H3K4me2 (Millipore 07-030; 1/200), mouse anti-CENP-A (AN1; 1/500), rabbit anti-CENP-C (R554; 1/500), mouse anti-RNAP II (phospho S2; 1/1,000) [H5] (abcam), mouse anti-H4K12ac (50B3/CMA412; 1/200), rabbit anti-H3K9ac (R607; 1/200), rabbit anti-H3K9me3 (abcam 8898; 1/500). Fluorophore-conjugated secondary antibodies were purchased from Jackson Labs. Marina Blue goat anti-rabbit secondary antibody (M-10992) was purchased from Life technologies and Alexa-405 donkey anti-mouse secondary antibody was purchased from abcam (ab175658).

The tetR-SNAP fusion proteins were detected by incubating the cells with either TMR-Star or SNAP-Cell Sir-647 (NEB) 30 min before fixation. CLIP-H3.3 was detected with benzylcytosine labelled with Alexa647 after fixation (NEB).

Preparation and staining of unfixed metaphase chromosomes was performed as previously described^16^. In brief, cells were arrested in metaphase with 150 ng ml^−1^ colcemid (KaryoMax, Gibco) for 3 h, and mitotic cells were collected by shake-off. Cells were subject to hypotonic treatment; cytospun on poly-lysine glass slides and incubated in KCM buffer (10 mM Tris pH 8.0; 120 mM KCl; 20 mM NaCl; 0.5 mM EDTA; 0.1% Triton X-100) for 10 min before labelling with antibodies in KCM buffer. After staining, samples were fixed in 4% Formaldehyde/KCM and mounted with Vectashield containing DAPI (Vector Labs).

Preparation of stretched mitotic chromatin fibres was performed as previously described^46^. In brief, cells were arrested in metaphase with 150 ng ml^−1^ colcemid (KaryoMax, Gibco) for 3 h, and mitotic cells were collected by shake-off. After incubation on hypotonic buffer and cytospun samples 10 min at 35 g on poly-lysine slides, cells were incubated in lysis buffer (2.5 mM Tris-HCl pH=7.5; 0.5 M NaCl; 1% Triton X-100; 0.4 M urea) for 20 min at RT. Samples were subsequently fixed in 4% Formaldehyde/1 × PBS solution and indirect immunofluorescence was performed following standard procedures. Bacterially purified tetR-EYFP fusion protein was incubated after the secondary antibodies diluted in a solution of 1% BSA/1 × PBS at RT.

### Image acquisition and fluorescence signal quantification

Microscope images were acquired on a DeltaVysion Core system (Applied Precision) using an inverted Olympus IX-71 stand, with an Olympus UPlanSApo × 100 oil-immersion objective (numerical aperture 1.4) and a 250 W Xenon light source. Camera (Photometrics Cool Snap HQ), shutter and stage were controlled through SoftWorx (Applied Precision). Z-series were collected with a spacing of 0.2 μm, and image stacks were subsequently deconvolved in SoftWorx.

Immunofluorescence signals in deconvolved images were analysed in ImageJ software (National Institutes of Health, Bethesda, MD). For CENP-A and CENP-C signal quantification, a custom-made macro in ImageJ modified from Bodor *et al.*^42^, was used. In brief, the CENP-A or CENP-C signal (Texas Red or Alexa647) at the HAC-associated EYFP signal was determined for every z-section within a 7 square pixel box. The mean signal intensity in the HAC section was obtained and the minimum intensity within the section was used for background subtraction. Average intensity of signals in endogenous centromeres was used as normalizer. For epigenetic marks signal quantification and CLIP-H3.3, an area determined by the HAC-associated EYFP signal was selected for quantification. Average signal for the epigenetic mark on the HAC area was determined and normalized for the average signal of the mark contained in HAC-flanking areas of the same size (endogenous levels). Background was subtracted for both HAC-associated signals and HAC-flanking signals.

### Pulse-chase experiments with SNAP and Halo-tagged CENP-A

Cells were co-transfected as described above with the relevant tetR-EYFP constructs and either pCENP-A-SNAP-IP or Halo-CENP-A constructs. In all, 16 h after transfection, existing CENP-A-SNAP was blocked with BG (New England Biolabs) following manufacturer's instructions. Existing Halo-CENP-A was rendered non-fluorescent with Biotin-conjugated Halo-Ligand (Promega). Newly synthesized CENP-A-SNAP or Halo-CENP-A were labelled after a chasing time of 7 h and 30 min using TMR-Star substrate (NEB) or Coumarin-conjugated Halo-ligand (Promega), respectively, for 20 min following manufacturer's instructions. Following the washes of unbound substrate, cells were fixed in 2.6% formaldehyde/1 × PBS, counterstained with Hoechst (SNAP experiments) and mounted with Vectashield (Vector Labs).

To analyse the stability of Halo-CENP-A molecules at centromeres, a pulse of 30 min with Coumarin-conjugated Halo-Ligand was performed 16 h after transfection. Following the washes of unbound substrate, cells were incubated for 24 h, fixed in 2.6% formaldehyde/1 × PBS and mounted with Vectashield without DAPI (Vector Labs).

Microscope images were acquired on a DeltaVysion Core system and the quantification of HAC-associated TMR-Star, Sir-Alexa647 or Coumarin Halo-ligand signals was done using ImageJ software as described before.

### ChIP experiments

Cell lysates were crosslinked in 1% formaldehyde and ChIP experiments were performed using a protocol described in detail in the Supplementary Methods section. The following monoclonal antibodies were used: anti-H3K4me2 (003), anti-H3K9ac (005), H3K36me2 (2C3) (ref. 65). Oligonucleotide primer pairs for RT-PCR are described below.

### Real-time RT-PCR analysis

Total RNA was extracted using TRIzol reagent (Invitrogen) according to manufacturer's instructions. In brief, 2 μg of RNA were converted to cDNA using SuperScript III Reverse Transcriptase (Invitrogene) following manufacturer's instructions with OligodT primers (Sigma). Real-time PCR analysis of cDNA equivalent to ≈40 ng (alphoid^tetO^, alphoid^chr21^) or ≈0.4 ng (Bsr, β-actin) input RNA was subsequently performed using a SYBR Green Mastermix (Roche) on a LightCycler480 system (Roche) and the following oligonucleotides: tetO-Fw (5′-CCACTCCCTATCAGTGATAGAGAA-3′) and either tetO-Rv (5′-TCGACTTCTGTTTAGTTCTGTGCG-3′) for the ChIP experiments or tetO-Rv2 (5′-GTTAAACTCAGTCGTCACCAAGAG-3′) for RNA experiments to detect the alphoid^tetO^ array, Chr21-Fw (5′-GTCTACCTTTTATTTGAATTCCCG-3′) and Chr21-Rv (5′-AGGGAATGTCTTCCCATAAAAACT-3′) for the alphoid^chr21^ array, bsr-Fw (5′-CAGGAGAAATCATTTCGGCAGTAC-3′) and bsr-Rv (5′-TCCATTCGAAACTGCACTACCA-3′) for the blasticidin resistance gene, sat2-Fw (5′-TCGCATAGAATCGAATGGAA-3′) and sat2-Rv (5′-GCATTCGAGTCCGTGGA-3′) for the pericentromeric alphoid^chr^ 1, act-Fw (5′-GCCGGGACCTGACTGACTAC-3′) and act-Rv (5′-AGGCTGGAAGAGTGCCTCAG-3′) for actin. For every oligonucleotide primer pair and every plate, a standard curve was created from genomic DNA derived from the 1C7 cell line. Background values (no reverse transcriptase) were subtracted, and all values were normalized to β-actin expression. The transcript levels were expressed relative to the +Dox values of the alphoid^tetO^ HAC, which was arbitrarily set to 100.

### Data availability

The data that support the findings of this work are available from the corresponding author on request.

## Additional information

**How to cite this article:** Molina, O. *et al.* Epigenetic engineering reveals a balance between histone modifications and transcription in kinetochore maintenance. *Nat. Commun.* **7**, 13334 doi: 10.1038/ncomms13334 (2016).

**Publisher's note:** Springer Nature remains neutral with regard to jurisdictional claims in published maps and institutional affiliations.

## Supplementary Material

Supplementary Information Supplementary Figures 1-7, Supplementary Tables 1-3 and Supplementary Methods

Peer Review
