# Supplementary material for: Epigenetic engineering reveals a balance between histone modifications and transcription in kinetochore maintenance
Source: Nat Commun. 2016 Nov 14;7:13334. doi: 10.1038/ncomms13334 (PMC5114538; doi:10.1038/ncomms13334)
Supplement: Supplementary Information — Supplementary Figures 1-7, Supplementary Tables 1-3 and Supplementary Methods [file ncomms13334-s1.pdf]

SUPPLEMENTARY INFORMATION

1. Supplementary Figures

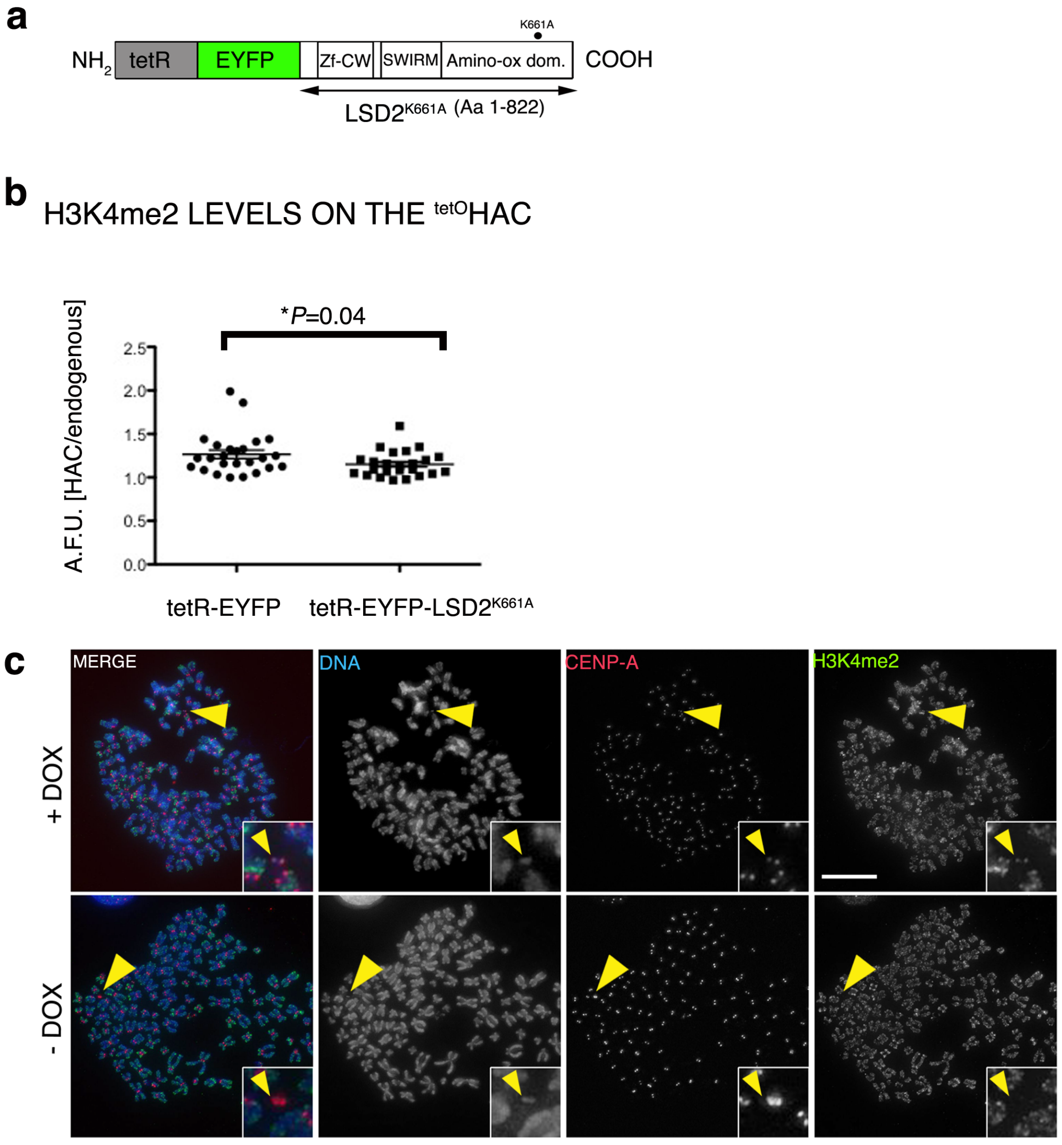

Supplementary Figure 1

Supplementary Figure 1. Tethering LSD2 to the alphoid^{tetO} HAC decreases the H3K4m2 levels (Related to Figure 1)**.** (**a**) Schematic drawing of the tetR-EYFP-LSD2^{K661A} mutant (**b**) Fluorescence signals of HAC-associated H3K4me2 quantification as in Figure 1 after transfection of tetR-EYFP-LSD2^{K661A} single mutant.

Tethering LDS2^{K661A} to the HAC slightly reduces H3K4me2 levels at the alphoid^{tetO} HAC centromere (*P*=0.04; Mann-Whitney test). Solid bars indicate the medians and error bars represent the standard error of the mean (s.e.m). N= two independent experiments. Asterisks indicate a significant difference (p<0.05; Mann-Whitney test). (**c**) Immunofluorescence analysis of unfixed metaphase chromosomes stained with indicated antibodies in the presence of doxycycline (+ DOX; *top*) and in after 24h of doxycycline washout (-DOX; *bottom*). Scale bar: 10 μm.

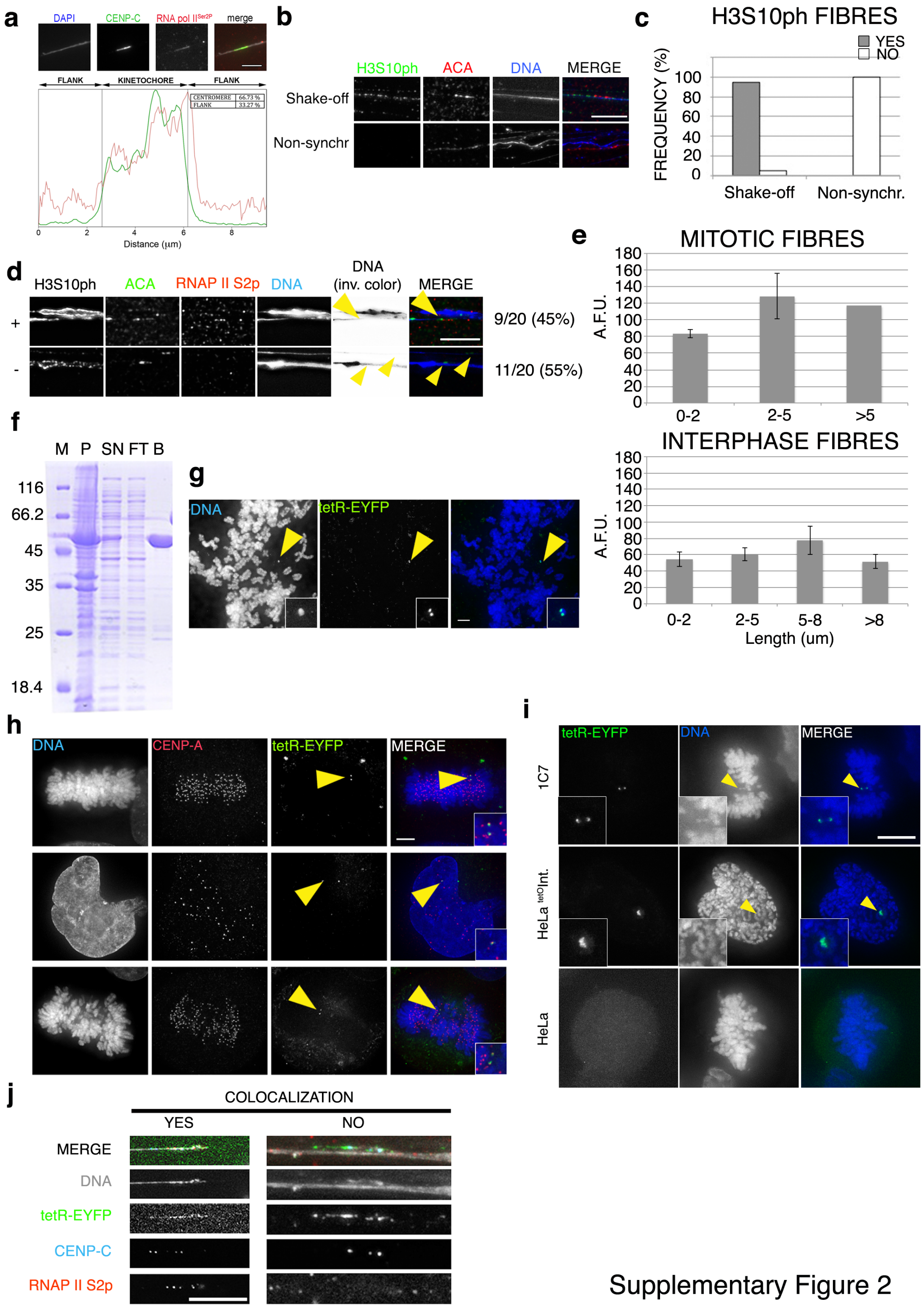

Supplementary Figure 2

Supplementary Figure 2. Active transcription at the kinetochore and detection of the alphoid^{tetO} HAC by *in situ* tethering tetR-EYFP (Related to Figure 2)**.** (**a**)

Immunofluorescence analysis of stretched chromatin fibers stained for CENP-C, RNAP II-S2ph and counterstained with DAPI. Representative line-scan fluorescence intensity graph showing the spread of RNAP II-S2ph signal on chromatin fibers at kinetochores as determined by the CENP-C signal (*bottom*). Table show the average of 10 informative fibers. (**b**) Representative images of chromatin fibers after mitotic shake-off (*top*) and unsynchronized cultures (*bottom*) stained with antibodies to H3S10ph (panel 1) or ACA (panel 2). (**c**) Quantification of the frequency of fibers containing H3S10ph signal (N=20 fibers after mitotic shake-off and 15 fibers from unsynchronized cultures of two independent experiments). (**d**) Representative images of mitotic chromatin fibers stained with H3S10ph (panel 1), ACA (panel 2) and RNAP II-S2ph (panel 3). DNA was counterstained with DAPI (panel 4). Inverted DAPI images (panel 5) were included to identify the individual fibers containing the IF signals. Merged images represent the overlay of ACA, RNAP II-S2ph and DAPI. Arrowheads depict centromeric fibers. Images show an example and the frequency of fibers with (*top*) and without (*bottom*) RNAP II-S2ph colocalization with ACA signals (N=20 fibers in two independent experiments). (**e**) Quantification of the length and the mean intensity of ACA signals from mitotic (*top*) and interphase (*bottom*) fibers (N=20 mitotic fibers and 15 interphase fibers of two independent experiments). Error bars represents the standard error of the mean (s.e.m). (**f**) Purification of TetR-EYFP. SDS-PAGE analysis of TetR-EYFP after Ni-NTA affinity purification. M: Marker (Pierce, 26610); P: Pellet (Insoluble fraction); SN: Supernatant (Soluble fraction); FT: Flow through (Unbound fraction); B: Pool of elutions. Molecular weight of TetR-EYFP: 52KDa. (**g**) Representative image showing the tetR-EYFP *in situ* tethering on 1C7 metaphase chromosome spreads. Arrowheads depict the HAC as determined by the EYFP and DAPI signals. (**h**) Representative images showing the tetR-EYFP *in situ* tethering on 1C7 cells stained with CENP-C antibody. Images show

examples of tetR-EYFP *in situ* tethering on metaphase (*top*), interphase (*middle*) and prometaphase (*bottom*) cells. (**i**) Representative images showing *in situ* tetR-EYFP tethering on 1C7 cells (*top*), HeLa3-8-Int (*middle*) and WT HeLa cells (negative control; *bottom*). (**j**) Additional examples of mitotic stretched chromatin fibers stained with tetR-EYFP, CENP-C and RNAP II-S2ph and DNA counterstained with DAPI. Images show co-localization of RNAP II-S2ph and CENP-C on the HAC (*left*) and a fiber without RNAP II-S2ph signal (*right*). Arrowheads depict the alphoid^{tetO} HAC as determined by the EYFP signal. Scale bars: 5 μm.

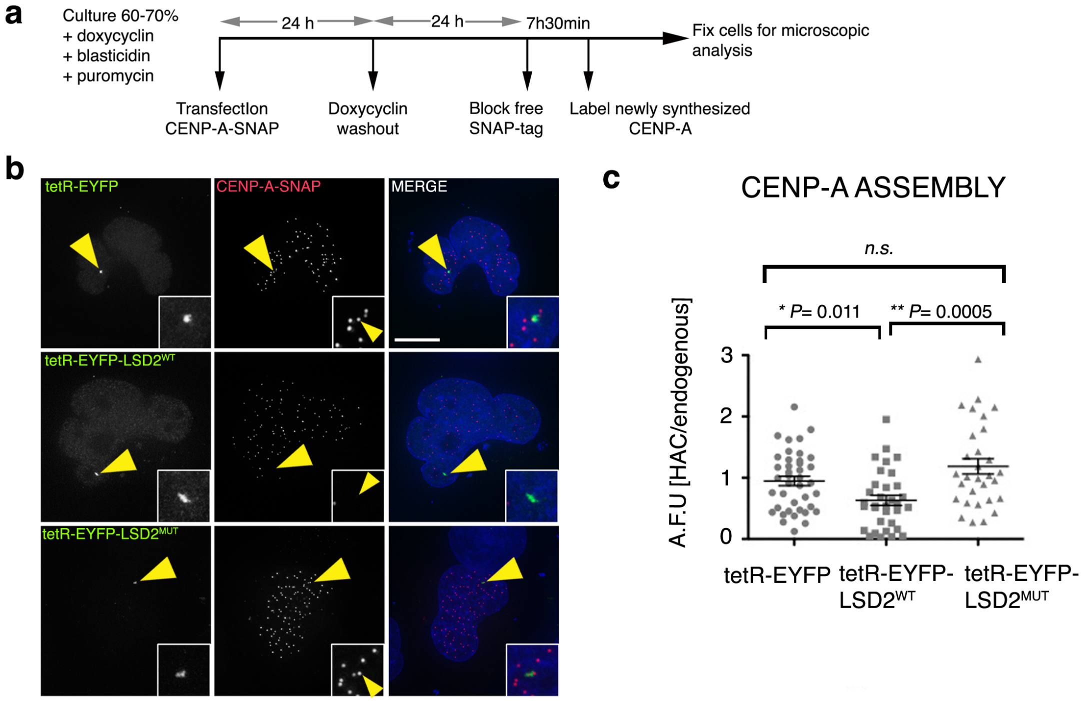

Supplementary Figure 3

Supplementary Figure 3. H3K4me2 removal affects the loading of new CENP-A molecules to the alphoid^{tetO} HAC centromere (Related to Figure 3)**.** (**a**) Schematic workflow to determine the incorporation of newly synthesized CENP-A at centromeres. (**b**) Representative images of CENP-A-SNAP analysis in 1C7 cells expressing the indicated tetR-EYFP fusion proteins. Scale bar= 10 µm (**c**) Quantification of TMR-Star fluorescence signal levels associated with the HAC and normalised to the average signal at endogenous centromeres. Solid bars indicate the medians and error bars represent the standard error of the mean (s.e.m). N= three independent experiments. Asterisks indicate a significant difference (*p<0.05; **p<0.01; Mann-Whitney test).

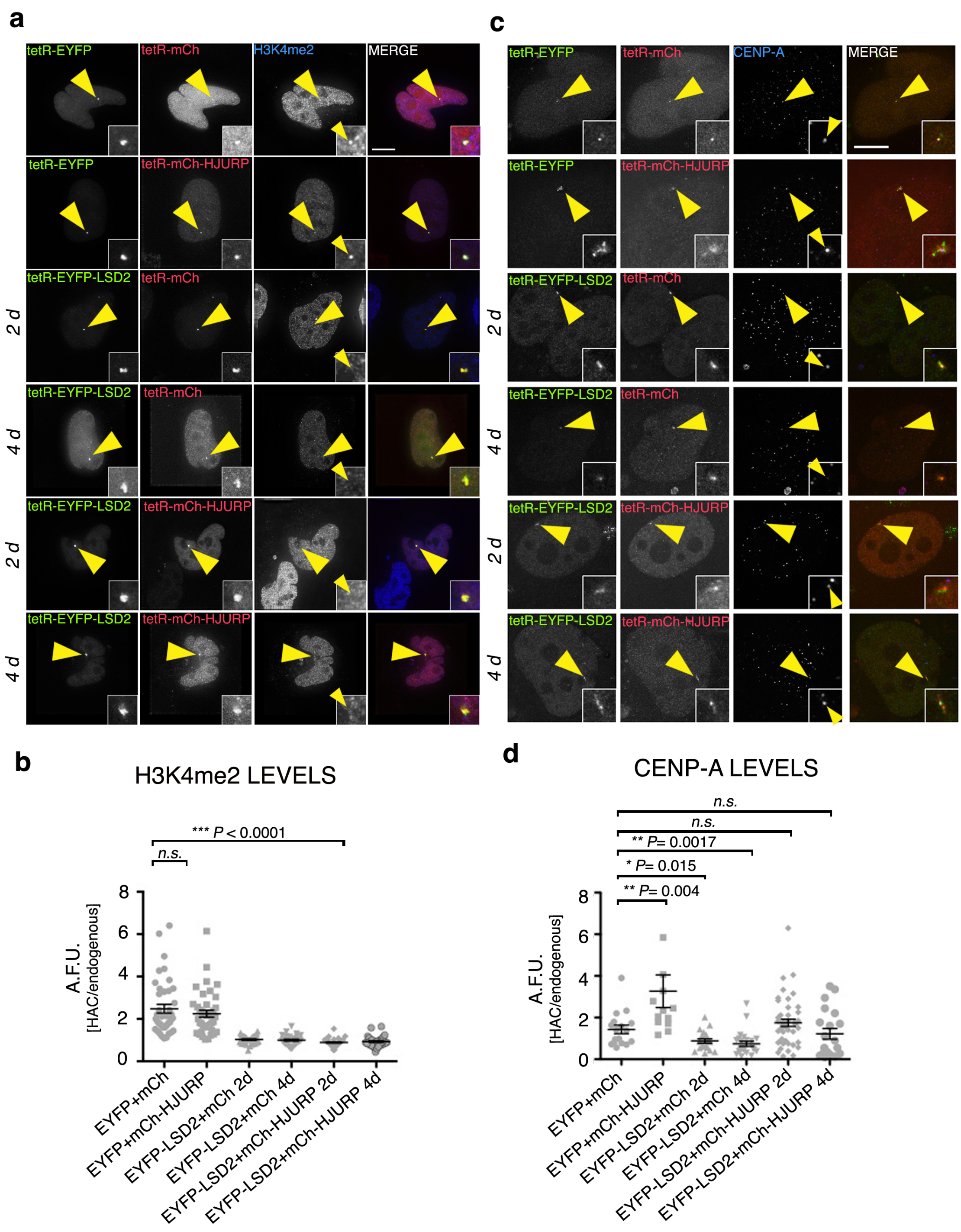

Supplementary Figure 4

Supplementary Figure 4. Tethering HJURP together with LSD2 to the alphoid^{tetO} HAC centromere confirms the reliability of the "*in situ* epistasis" assay (Related to Figure 5). (**a**) Representative immunofluorescence (IF) images of 1C7 cells expressing the indicated tetR-fusion proteins at the indicated time-points

and staining for H3K4me2. (**b**) Fluorescence signals of HAC-associated H3K4me2 staining in individual cells transfected as in A were quantified and plotted as A.F.U. (**c**) Representative IF images of 1C7 cells expressing the indicated tetR- fusion proteins at the indicated time points and stained for CENP-A. (**d**) Quantification of fluorescence signals of HAC-associated CENP-A. Solid bars indicate the medians and error bars represent the standard error of the mean (s.e.m). N= two independent experiments per time point and staining. Asterisks indicate a significant difference (* $p<0.05$; ** $p<0.01$; Mann-Whitney test). Scale bars: 10 µm.

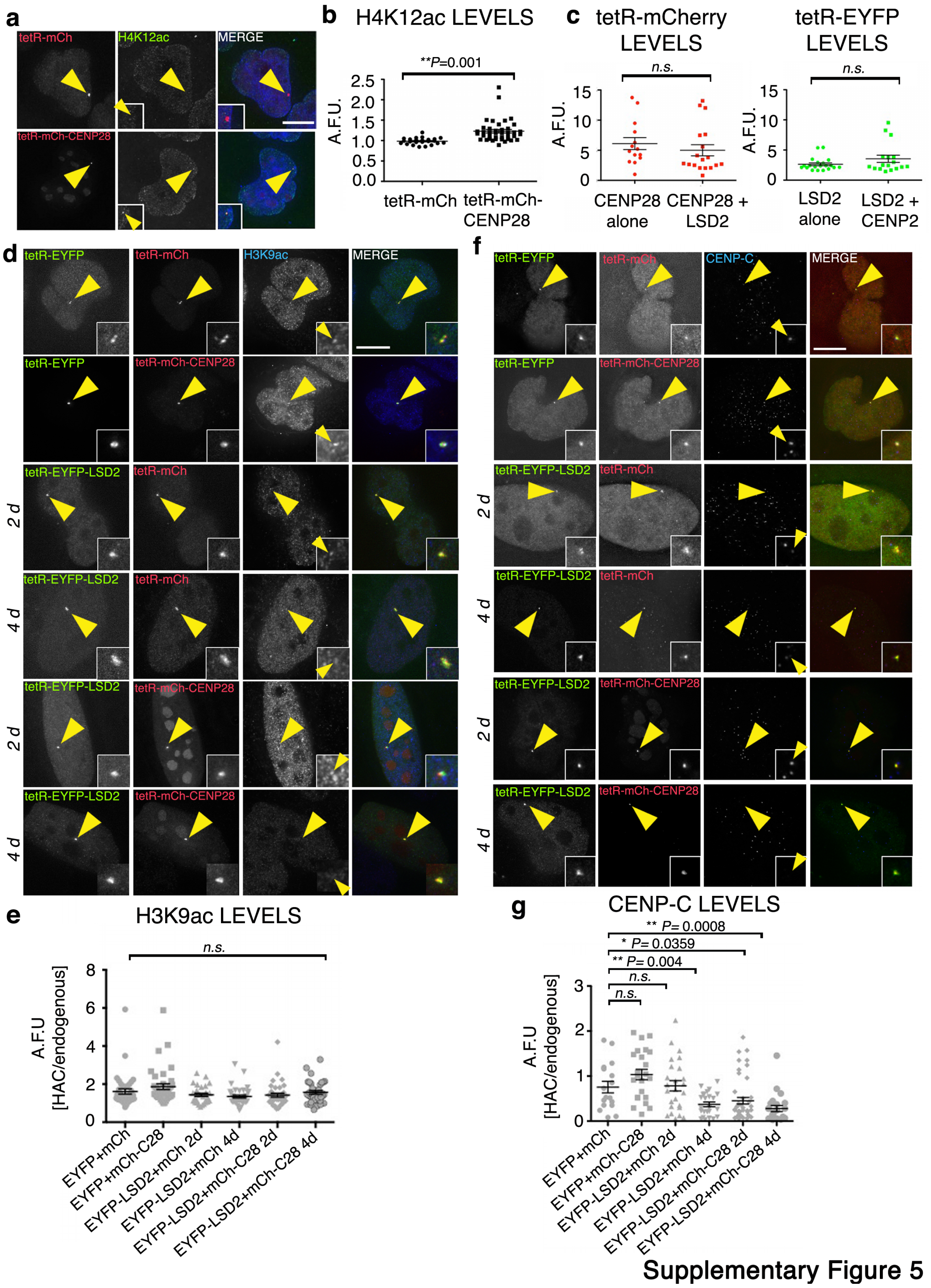

Supplementary Figure 5. Tethering a transcriptional activator to the alphoid^{tetO} HAC centromere is not sufficient for kinetochore maintenance in the absence of H3K4me2 (Related to Figures 5)**.** (**a**) Representative IF images of 1C7 cells expressing the indicated tetR-fusion proteins for 24 hours and staining for H4K12ac

(panel 2). Arrowheads depict the HAC, as determined by the mCherry signal (panel 1). Merged images (panel 3) represent the overlay of mCherry signals with antibody and DAPI. (**b**) Fluorescence signals of HAC-associated H4K12ac staining in individual cells transfected as in A were quantified and plotted as A.F.U. Solid bars indicate the medians and error bars represent the standard error of the mean (s.e.m). Tethering CENP-28 to the HAC significantly increases the levels of H4K12ac at the alphoid^{tetO} HAC centromere within 24 h (P=0.001; Mann-Whitney test). (**c)** Quantification of the levels of tetR-mCherry (*left*) and tetR-EYFP (*right*) fusion proteins on the alphoid^{tetO} HAC plotted as A.F.U. Solid bars indicate the medians and error bars represent the standard error of the mean (s.e.m). (**d**) Representative IF images of 1C7 cells expressing the indicated tetR-EYFP and tetR-mCherry fusion proteins (panel 1 and 2) at the indicated time points and stained for H3K9ac (panel 3). Merged images represent the overlay of EYFP and mCherry signals with H3K9ac antibody (panel 4). (**e**) Fluorescence signals of HAC-associated H3K9ac staining in individual cells transfected as in D were quantified and plotted as A.F.U. (**f**) Representative IF images of 1C7 cells expressing the indicated tetR-EYFP and tetR-mCherry fusion proteins (panel 1 and 2) at the indicated time points and stained for CENP-C (panel 3). Merged images represent the overlay of EYFP and mCherry signals with CENP-C antibody (panel 4). (**g**) Fluorescence signals of HAC-associated CENP-C staining in individual cells transfected as in F were quantified and plotted as A.F.U. Solid bars indicate the medians and error bars represent the standard error of the mean (s.e.m). N= two independent experiments per time point and staining. Asterisks indicate a significant difference (*p<0.05; **p<0.01; Mann-Whitney test). Scale bars: 10 µm.

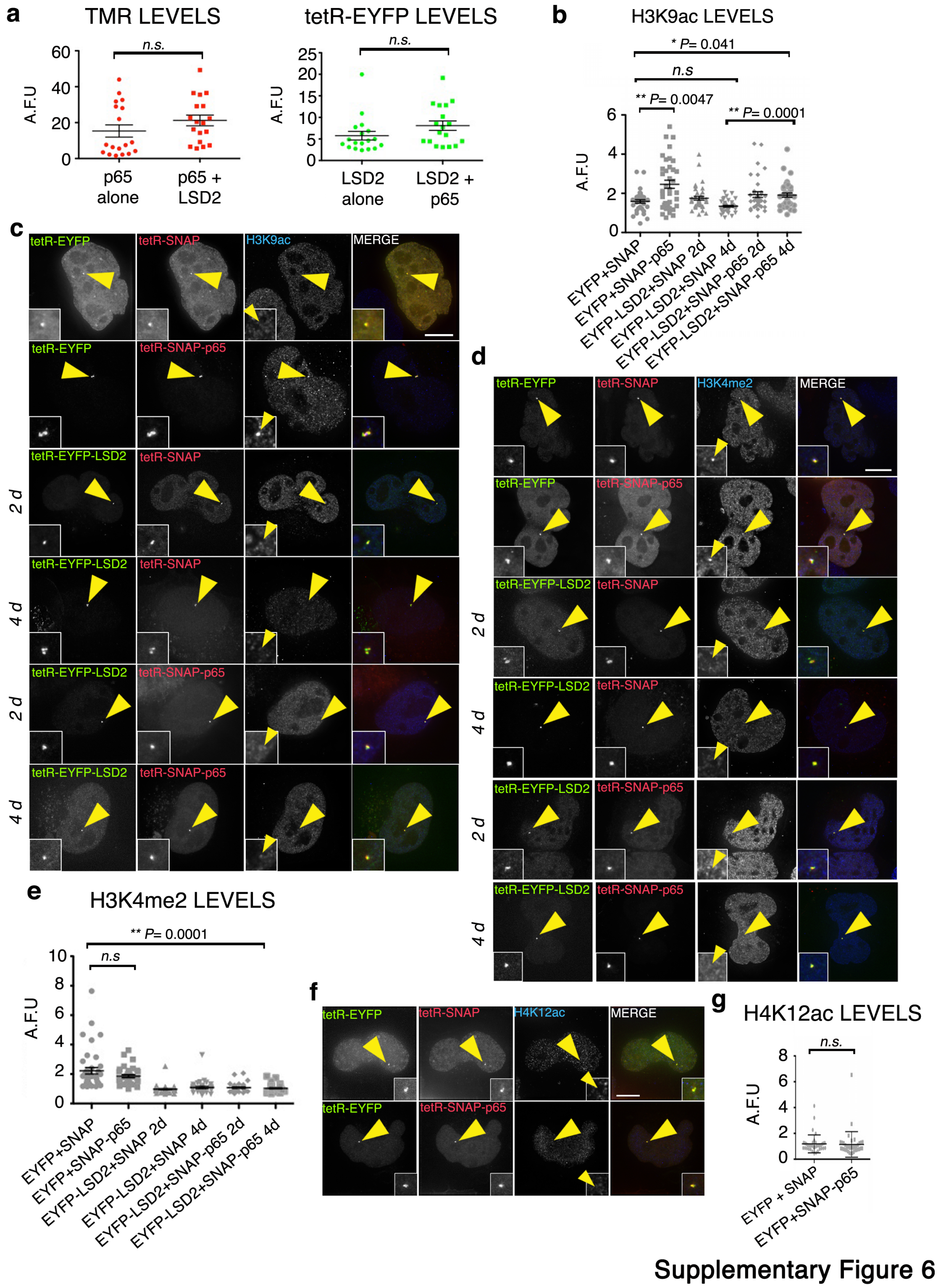

Supplementary Figure 6. Tethering tetR-SNAP-p65 to the alphoid^{tetO} HAC bypasses the requirement for H3K4me2 on kinetochore maintenance. (Related to Figure 6). (**a**) Quantification of the levels of tetR-SNAP (detected with TMR; *left*) and tetR-EYFP (*right*) fusion proteins on the alphoid^{tetO} HAC plotted as A.F.U. Solid

bars indicate the medians and error bars represent the standard error of the mean (s.e.m). (**b**) Quantification of the HAC-associated H3K9ac signals in individual cells transfected as in C and plotted as A.F.U. Representative immunofluorescence (IF) images of 1C7 cells expressing the indicated tetR-fusion proteins at the indicated time-points and staining for H3K9ac (**c**) and H3K4me 2 (**d**). (**e**) Fluorescence signals of HAC-associated H3K4me2 staining in individual cells transfected as in D were quantified and plotted as A.F.U. (**f**) Representative IF images of 1C7 cells expressing the indicated tetR-fusion proteins at the indicated time-points and staining for H4K12ac. (**g**) Quantification of the HAC-associated H3K9ac signals in individual cells transfected as in F and plotted as A.F.U. Solid bars indicate the medians and error bars represent the standard error of the mean (s.e.m). N= two independent experiments per time point and staining. Asterisks indicate a significant difference (*$p<0.05$; **$p<0.01$; Mann-Whitney test). Scale bars: 10 µm.

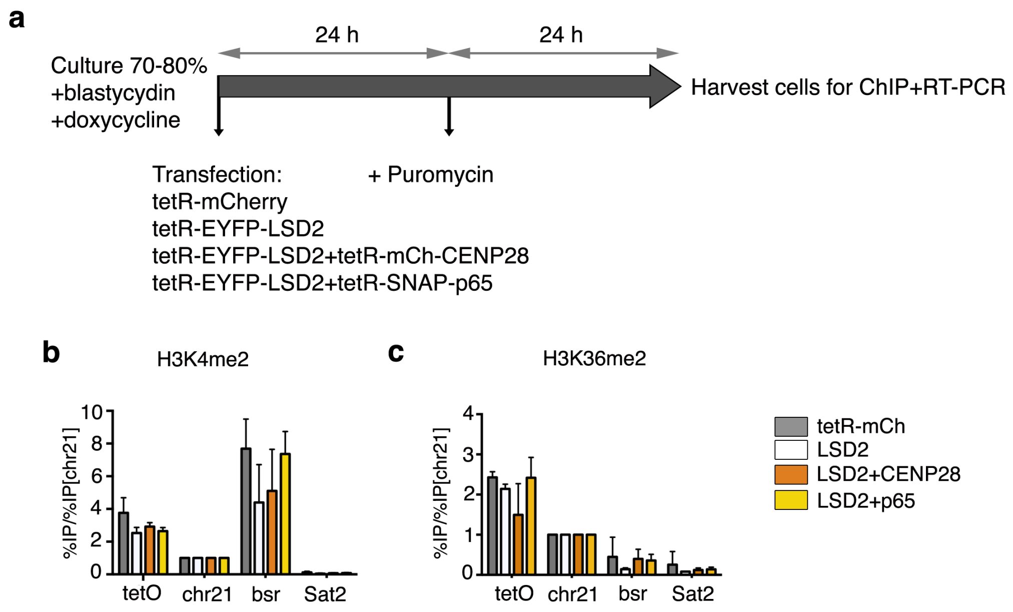

Supplementary Figure 7

Supplementary Figure 7. H3K4me2 and H3K9ac modifications maintain the epigenetic signature of centrochromatin for centromere stability. (Related to Figure 8). (**a**) Schematic workflow to perform ChIP experiments after transient expression of the indicated tetR-fusion proteins. (**b** and **c**) RT-PCR analysis of ChIPed DNA from 1C7 cells expressing the indicated tetR-fusion proteins for H3K4me2 (**b**), H3K36me2 (**c**). Values represent the levels of the indicated epigenetic marks normalized to the values observed for the chromosome 21 centromere. Data show the mean and s.d. of three independent experiments.

2. Supplementary Tables

Supplementary Table 1. RT-PCR results to detect centromeric transcripts. Cp values (normalised for β-actine) are relative to the internal control, which was arbitrary set as 100. The average of the three independent experiments for each situation analysed and the *P*-value comparing with the respective internal control are shown.

Experiment	Replicate	Cp value tetO/actin	Average (±SEM)	*P*-value
LSD2	1	25.97	32.44 ± 17.909	0.01956 *
	2	5.16		
	3	66.18		
$LSD2^{MUT}$	1	189.21	100.363 ± 54.229	0.99497
	2	109.81		
	3	2.0688		
LSD2+CENP-28	1	228.15	258.19 ± 15.018	0.000459 ***
	2	273.21		
	3	219.62		
LSD2+P65	1	192.52	201.578 ± 15.255	0.002642 **
	2	180.87		
	3	231.34		

Supplementary Table 2. RT-PCR results to detect centromeric transcripts on mitotic cells. Cp values (normalised for β-actine) are relative to the control (tetR-EYFP), which was arbitrary set as 100. The average of the three independent experiments for each situation analysed and the *P*-value comparing with the respective internal control are shown.

Experiment	Replicate	Cp value tetO/actin	Average (±SEM)	P-value
LSD2	1	24.82	47.437 (± 17.009)	0.036664*
	2	86.75		
	3	36.73		
LSD2+CENP-28	1	217.64	401.213 (± 101.401)	0.041123*
	2	418.35		
	3	567.65		
LSD2+P65	1	179.87	244.816 (± 39.789)	0.021972*
	2	237.46		
	3	317.11		

Supplementary Table 3. Primers used for constructing tetR-fusion proteins and restriction sites included in the sequence (RS).

PRIMER NAME	SEQUENCE (5'-3')	RS
LSD2_Fw	ATACAGATCTATGGCAACTCCACGGGGGAGG	BglII
LSD2_Rv	GCCGGCGTTAAAATGCTGCAATCTTGCTTGCTTC	NotI
mCh_Fw	ACCGGTAATGGTGAGCAAGGGCGAGGAGG	AgeI
mCh_Rv	GGCGCGCCGGTACAGCTCGTCCATGCCGCCG	AscI
C28_Fw	GGCGCGCCATGGCGATGCACAACAAGGCGG	AscI
C28_Rv	GCGGCCGCCTAATAGTCAGCTCGTGG	NotI
mChHJ_Fw	AGCGCTATATGGTGAGCAAGGGCGAGGAG	AfeI
mChHJ_Rv	CTCGAGGTGGTACAGCTCGTCCATGCCG	XhoI
SNAP_Fw	ACCGGTAATGGACAAAGACTGC	AfeI
SNAP_Rv	TGTACATTGCAGGACCCAGCCCAGGCTTG	BsrGI

3. Supplementary Methods

Construction of tetR fusion constructs

The tetR-EYFP-LSD2^{WT} constructs were obtained as follows. The coding sequence of full-length human LSD2 (NCBI accession number: 221656) in pcDNA3-GFP was a kind gift from Dr. Y. Geno Shi (Harvard Medical School; Boston; MA 02115). LSD2 was PCR amplified using the oligonucleotides LSD2_Fw and LSD2-Rv (Supplementary Table 3). The PCR product was digested with BglII and NotI endonucleases and cloned into the BamHI and NotI restriction sites of tYIP vector [30] to generate tYIP-LSD2, expressing tetR-EYFP-LSD2^{WT} from a CMV promoter and conferring resistance to Puromycin. To generate the E412AK661A double mutant the tYIP-LSD2 construct was subjected to a double round of site-directed mutagenesis using the QuikChange II kit (Stratagene).

The tYIP vector was modified to express tetR-mCherry fusion proteins by replacing EYFP for the mCherry gene. mCherry gene in pmCherry vector (Clontech) was PCR amplified using the oligonucleotides mCh_Fw and mCh_Rv (Supplementary Table 3). The PCR product was digested with AgeI and AscI endonucleases and cloned in the AgeI and AscI restriction sites of tYIP vector, replacing the EYFP gene for mCherry and thus generating the tYIP-mCh targeting vector. The coding sequence of CENP-28 (NCBI accession number GI:400154070) was PCR amplified from the tYIP vector using the oligonucleotides C28_Fw and C28_Rv (Supplementary Table 3). The PCR product for CENP-28 was digested with AscI and NotI endonucleases and cloned into the same sites into the tYIP-mCh vector to generate tYIP-mCherry-CENP-28 construct. The tetR-mCherry-HJURP vector was constructed by replacing the EYFP gene for the mCherry gene from the pJETY-tetR-EYFP-HJURP construct. mCherry was amplified using the

oligonucleotides mChHJ_Fw and mChHJ_Rv (Supplementary Table 3). The PCR product was digested and cloned in the AfeI and XhoI restriction sites of the pJETY-tetR-EYFP-HJURP vector, thus generating pJETY-tetR-mCherry-HJURP.

The tetR-SNAP-p65 vector was constructed by replacing the EYFP gene for the SNAP tag gene from the tYIP-tetR-EYFP-p65 construct [31]. The SNAP tag coding sequence was amplified using the oligonucleotides SNAP_Fw and SNAP_Rv (Supplementary Table 3). The PCR product was digested and cloned in the AgeI and BsrGI sites of the tYIP-tetR-EYFP-p65 construct, thus generating tYIP-tetR-SNAP-p65 construct. To generate the tYIP-tetR-SNAP construct, we removed the p65 gene by digesting the tYIP-SNAP-p65 construct with the BsrGI and BamHI endonucleases and re-ligating the vector after end-blunting using the T4 DNA polymerase (NEB).

Chromatin Immunoprecipitation (ChIP) experiments

Exponentially growing cells were washed in D-PBS (Gibco) and subsequently harvested with TrypLE Express (Gibco). Cells were ressuspended in D-PBS up to a concentration of $1x10^6$ cells/ml and crosslinked in a final 1% Formaldehide solution (Sigma) for 5 minutes at room temperature, followed by quenching with 2.5M Glycine for 5 minutes at room temperature. Cells at a concentration of $5x10^6$ cells/ml were lysed in lysis buffer (10mM Tris pH=8.0; 10mM NaCl; 0.5% NP-40) containing protease inhibitors (1 µg/ml CLAP; 0.5 µg/ml Aprotinin; 1mM PMSF) for 10 min on ice. Nuclei were briefly washed in lysis buffer with protease inhibitors in 300 µl of Dilution Buffer 1 (50 mM Tris pH=8.0; 2 mM EDTA; 0.2% SDS; 134 mM NaCl; 0.88% Triton X-100; 0.088% Na-deoxycholate). Chromatin was sheared by sonication in a Bioruptor sonicator (Diagenode) for 14 cycles (30s ON/ 30S OFF) at high setting and 4°C. Supernatant products of sonication were diluted with 300 µl of Dilution Buffer 1, 500 µl of Dilution Buffer 2 (50 mM Tris pH=8.0; 167 mM NaCl; 1.1% Triton X-100;

0.11% Na-deoxycholate) and 500 µl of RIPA buffer containing 150 µl of NaCl (RIPA-150) and protease inhibitors. Antimouse IgG Dynabeads (Invitrogen) were coated with the relevant antibodies for 6 hours with RIPA-150/0.5% BSA at 4°C, washed twice with RIPA-150/0.5% BSA and 500 µl of sheared chromatin was incubated with the beads at 4°C overnight. Beads were afterwards washed twice with RIPA-150 and RIPA buffer containing 500 mM of NaCl (RIPA-500) and a final wash with TE pH=8.0. Antibody/chromatin complexes were decrosslinked with 10% Chelex-100 resin (BioRad) in water at 93 ^{o}C and treated with RNase A and Proteinase K. DNA was subsequently recovered by pipetting out 60 µl of the supernatant in a new eppendorf tube.

ChIPed DNA was subjected to RT-PCR using a SYBR Green Master Mix (Roche) as previously decribed (Cardinale et al., 2009).

Expression and purification of recombinant TetR-EYFP.

TetR was cloned into a pET23a vector as a C-terminally His-tagged protein. The pET23a-TetR-eYFP vector was transformed into *E. coli* BL21 Gold competent cells and grown at 37°C in Super Broth-medium containing ampicillin until OD_{600} reached 1. The culture was induced overnight at 18°C with IPTG at a final concentration of 0.35 mM. Cells were lysed in a buffer containing 20 mM Tris–HCl pH 7.5, 500 mM NaCl, 35 mM imidazole and 2 mM 2-mercaptoethanol. The TetR-eYFP protein was purified by affinity chromatography using a Ni-NTA column (GE Healthcare). The protein-bound column was washed with 20 mM Tris–HCl pH 7.5, 500 mM NaCl, 35 mM imidazole and 2 mM 2-mercaptoethanol followed by 20 mM Tris–HCl pH 7.5, 1000 mM NaCl, 50 mM KCl, 10 mM $MgCl_2$, 2 mM ATP, 35 mM imidazole and 2 mM 2-mercaptoethanol. The protein was eluted with 20 mM Tris–HCl pH 7.5, 150 mM NaCl, 400 mM imidazole and 2 mM 2-mercaptoethanol. The pooled eluted fractions

were dialysed overnight against 20 mM Tris–HCl pH 7.5, 150 mM NaCl, 5% glycerol and 2 mM 2-mercaptoethanol. The purity of the sample was analysed by 15% SDS-PAGE stained with Coomassie Blue.
