# Supplementary material for: Epigenetic engineering reveals a balance between histone modifications and transcription in kinetochore maintenance
Source: Nat Commun. 2016 Nov 14;7:13334. doi: 10.1038/ncomms13334 (PMC5114538; doi:10.1038/ncomms13334)
Supplement: Peer Review [file ncomms13334-s2.pdf]

PEER REVIEW FILE

Reviewers' comments:

Reviewer #1 (Remarks to the Author):

Review of Molina et al.

In this study the authors use human artificial chromosome (HACs) to investigate how modifying epigenetic marks in centromeric chromatin influence segregation functions, and the relationship with transcription in centromeric chromatin. They cleverly utilize tetO sequences on the HACs to target individual factors, or combinations of factors, that alter the histone modifications and/or transcriptional status, specifically LSD2 (H3K4me2 demethylase), the C-terminal activating domain of the NFKB p65 subunit (increases H3K9ac and transcription), and CENP-28 /Eaf6 (increases H4K12ac). One of the exciting applications of this technology is the demonstration that two different factors can be successfully targeted to the same HAC alphoid arrays ('in situ epistasis'), which allows the contributions of different modifications and centromeric transcription to be deconvolved. From these analyses the authors conclude that loss of H3K4me2 results in less transcription from centromeric regions, defective new assembly and long-term stability of the centromere specific histone CENP-A (using SNAP tags), and aberrant enrichment for the heterochromatin mark H3K9me3 at the centromere. However, inducing transcription by an alternative method, namely CENP28/Eaf-mediated elevation of H4K12ac could not rescue the defects associated with loss of H3K4me2, while increasing H3K9ac levels and transcript levels by targeting p65 did rescue the defects. The authors conclude that transcription alone is not sufficient for centromere propagation and function. They propose that transcription combined with the H3K9ac modification is sufficient for new CENP-A assembly, and is necessary to prevent spreading of heterochromatin/H3K9me3 spreading into the centromere.

Overall, this is a well-conceived and executed study that provides important new information about epigenetic regulation of centromere assembly, maintenance and function. However, I have some concerns about the chromatin fiber experiments, as well as the validity of some of the conclusions. Nevertheless, I heartily recommend publication in Nature Communications once the following issues have been addressed.

My only technical concern is with the chromatin fibers. The images presented show very strong DNA staining which is likely to represent bundles of fibers. Previous publications using this method rely on analyzing fibers that have very little visible DNA staining, to avoid

misinterpretations about the chromatin composition of specific genomic regions. In this case the specific concern is that conclusions about RNApol2 enrichment at centromeres could arise from the bundling of non-centromeric fibers that contain pol2 with centromeric fibers; in other words, there may be no pol2 on the centromeric chromatin, and results related to the presence of less pol2 after H3K4me2 removal are also called into question. This could also explain why many, but not all 'centromeric' fibers contain pol2. I suggest reanalyzing this data by first screening for tetO signals, then removing fibers that have strong DNA staining from the analysis. A related but more minor issue is whether or not these are truly mitotic fibers (see comment below), which could be addressed by incorporating staining for phosphorylated H3 ser10, a mitotic marker that should be retained on the fibers.

Examples of conclusions that go beyond the evidence presented:

page 9 bottom: "These data, together with previous results from our group 17 strongly suggest that the CENP-A chaperone HJURP requires H3K4me2 to load new CENP-A molecules at centromeres."
I do not question the result that H3K4me2 is important for new CENP-A assembly, but although HJURP is a key regulator of assembly, the data does not address if HJURP interacts directly with this mark. H3K4me2 could be more directly involved with other proteins or other parts of the assembly pathway. I would change it to '...suggest that new CENP-A assembly requires H3K4me2.", or include text that describes the logic more directly.

In the discussion and elsewhere in the manuscript a role for mitotic transcription is emphasized, but it is unclear how the authors are able to differentiate the functional impact of mitotic versus interphase transcription, both of which are claimed to occur at centromeres. Although it is appealing to imagine that the relevant transcription occurs in mitosis, just prior to the onset of new CENP-A assembly, given the evidence presented here and elsewhere it seems equally likely that interphase transcription (at least G2 and S) could be responsible for setting up 'assembly-competent' centromeric chromatin, even if assembly doesn't occur until late mitosis/G1.

minor issues:

p 19: "Importantly, all effects observed on CENP-A levels are corroborated by the effects observed on CENP-C in independent experiments."
This statement is not consistent with the following from p 11, reporting results from tethering CENP28/Eaf and observing increases in CENP-A.
"Interestingly, no corresponding increase was observed in the levels of CENP-C at the alphoidtetO HAC centromere (Figure S5F, G)."

p 15 top: Although it is clear that p65 tethering results in increased H3K9ac but not H4K12ac, I am confused as to why H3K9ac levels were not increased after tethering of CENP28/Eaf, which also increases the levels of centromeric transcripts? I understand that CENP28/Eaf is a H4K12-specific HAT, but would have expected H3K9ac to increase in response to transcription. What am I missing here?

p 18: "It has recently been reported that H3K4me2 defines transcription factor binding regions that overlap with promoters or enhancers 49 ."
Although this is true, it is important to note that H3K4me2 is highly enriched over transcribing gene bodies, not just promoters or enhancers, and cannot by itself be used to identify a role for promoters or enhancers, as implied here.

Reviewer #2 (Remarks to the Author):

Recent studies have pointed to the importance of active α-satellite RNA transcription and its associated active chromatin marks in directing CENPA deposition by HJURP at the centromere. However, it is yet not fully understood how these epigenetic marks facilitate centromere/CENPA chromatin assembly.

Earnshaw and colleagues have previously demonstrated that H3K4me2 and H3K36me3 are required for maintenance of centromere chromatin. They showed that targeting lysine-specific demethylase 1 (LSD1) led to depleted H3K4me2, resulting in reduced transcription of α-satellite DNA and ability of HJURP/CENPA targeting. In this study, they set to investigate the possible different roles of RNA transcription and active chromatin marks in directing centromere chromatin assembly. They tethered another KMT i.e. LSD2 to the alphoid satellite array on human artificial chromosome (HAC) and found loss of H3K4me2, transcription of α-satellite DNA and HJURP/CENPA targeting. They also investigated the impact of targeting H3K9 acetylation and H4K12 acetylation, in combination with the targeting of LSD2 to the alphoid array on HAC. The 'in situ epistatis' analysis is a good system as it allows the interrogation of how different histone modifications may interact to determine centromere chromatin identity, and pointed by the authors, allows them to uncouple transcription from specific histone modifications. Based on their findings, they conclude that " a balance of mitotic transcription, epigenetic modifications and chromatin remodelling in centrochromatin act as a barrier to prevent heterochromatin spreading and kinetochore inactivation in human centromeres".

In my opinion, the conclusions drawn from the current study are not entirely novel, and are not

fully supported by their data. Their previous studies had shown that tethering of the repressive heterochromatin factors caused loss of HAC centromeric transcription and inactivation of its centromere (Cardinale et al., 2009, Nakano et al., 2008, Bergmann et al., 2011, Ohzeki et al., 2012). The concept that the presence of active chromatin marks is required to maintain centromere transcription, CENPA chromatin assembly is not entirely novel.
The H3K9Ac/H3K9Me antagonistic balance for CENP-A maintenance is not new, in fact the authors had covered it their previous studies (Ozheki et al 2012 and Bergman et al 2012 etc). This detracts from the novelty of the study.

Despite this, I agree with the authors the importance of investigating the potential different functions of histone modifications (H3K4me2) and RNA transcription in directing centromere chromatin assembly, however, I am not convinced by their data that transcription is not important for CENPA chromatin assembly. As a whole I think this paper have a great potential and the authors have a great system work with, however, further experiments are needed to support their conclusion and there are major areas that need to be addressed:

1) The authors showed that expression of p65 which increases H3K9Ac (even in the LSD co-expressing cells, thus having low H3K4me2) resulted in an increase of transcription besides the increase in CENP-A loading. So it is not just specific histone modifications, but also the RNA transcription (Fig 6) that is required for CENPA chromatin assembly.

Targeting of p65 increases H3K9Ac, thus likely also directly prevents the formation of H3K9me3 chromatin. This also agrees with the idea that "...H3K9As directly/indirectly antagonises heterochromatin spreading into centrochromatin" (line 419- 420, p17). Fang et al 2010 showed that LSD2 can direct H3K9 methylation by G9a KMT. The inability of the increase in H4K12Ac and active transcription in CENP28 expressing cells to direct CENPA loading or centromere chromatin assembly may be because H3K9me (due to LSD2 targeting) is enriched at HAC centromere in both LSD2, LSD2/cenp28 expressing cells. Whereas, in LSD2/p65 expressing cells, the increase in H3K9Ac by p65 can antagonise the formation of H3K9me3 chromatin. Furthermore, p65 targeting also results in the increase in transcription at HAC centromere. The conclusion that p65 induced H3K9Ac, but not cenp28 induced H4K12Ac is sufficient to bypass the requirement of H3K4me2 is not entirely correct. It is most likely that both H3K9Ac (and the lack of H3K9me3) and transcription (promoted by H3K4me2 or H4K12Ac) are required for CENPA chromatin assembly.

2) Targeting p65 increased RNA transcription at the HAC centromere in LSD2 tarted cells, but not in CENPA loading. Would the authors look into the presence of H4K20me1 in LSD2, LSD/p65, LSD2/CENP28 expressing cells. They have shown that H4K20me2 is essential in CENP-A chromatin assembly (Hori et al 2014 Dev Cell). Does targeting of p65 affects

H4K20me1 formation, thus resulting in a reduced ability of CENPA chromatin assembly even with restored RNA transcription activity? Also what about H3K36me3 which is a slo found to be associated with centromere transcription by a previous study (Bergman et al 2012).

3) Re: pg 9, line 222-224, Could authors show changes in HJURP levels in the LSD2, LSD2/p65, LSD2/cenp-28 targeted cells? Their previous studies showed that H3K4me2 being important for HJURP recruitment (Bergmann et al 2011). Since CENP-A levels are also increased in LSD2/p65 (so high H3K9AC and low H3K4me2) expressing cells. This would clear up the uncertainty if it's just CENP-A loading and/or stabilisation that is affected. This is important as CENP-A chromatin propagation is a multistep complex pathway: priming of chromatin, removal of placeholders, recruitment of CENP-A loaders, loading of CENP-A, and stabilisation (i.e RSF and more importantly HATs required for Mis18, but insufficient for HJURP etc see Ohzeki 2012). Many of these other steps were not investigated in the current study.

4) Was level of CENPC looked at in cells expressing LSD2/cenp28 (Fig 5)?

5) A significant loss of transcripts is noted after 2 days of LSD2 expression, but RNA Pol2 and H3K4me2 levels only became significantly reduced after more than 4 days? Similar observation was made with CENP-A and C levels. Could this indicate that transcription and H3k4me2 are somewhat uncoupled and serve separate function. Perhaps transcription may be responsible for remodelling/priming the chromatin initially and also the reduction of transcripts may have a role in remodelling the chromatin as well (as suggested previously by Quenet 2014 eLife).

6) Fig 8 showed a greater increase of H3.3 loading in LSD2/cenp28 expressing cells. This increase in H3.3 loading also correlates with the higher RNA transcription in these cells (nearly 4 fold increase), when compared to that in LSD2/p65 expressing cells. Is it possible that the increase in H4k12Ac promotes a RNA pol2 activity that is too high, hence, affecting the loading/stabilisation of CENPA. Only a low level of transcription at the centromeres, a high level of RNA Pol2 may not be compatible with the maintenance of CENPA stability. Could authors consider modifying RNA pol2 targeting or activity to the HAC centromere using their system, without affecting the presence of H3K9Ac and/or H3K4me2? This would be useful to determine if RNA transcription is essential for CENPA loading and in other steps (priming, stabilisation, RSF positiioning etc.

Response to referees:

Reviewer #1 (Remarks to the Author):

Review of Molina et al.

In this study the authors use human artificial chromosome (HACs) to investigate how modifying epigenetic marks in centromeric chromatin influence segregation functions, and the relationship with transcription in centromeric chromatin. They cleverly utilize tetO sequences on the HACs to target individual factors, or combinations of factors, that alter the histone modifications and/or transcriptional status, specifically LSD2 (H3K4me2 demethylase), the C-terminal activating domain of the NFKB p65 subunit (increases H3K9ac and transcription), and CENP-28 /Eaf6 (increases H4K12ac). One of the exciting applications of this technology is the demonstration that two different factors can be successfully targeted to the same HAC alphoid arrays ('in situ epistasis'), which allows the contributions of different modifications and centromeric transcription to be deconvolved. From these analyses the authors conclude that loss of H3K4me2 results in less transcription from centromeric regions,
defective new assembly and long-term stability of the centromere specific histone CENP-A (using SNAP tags), and aberrant enrichment for the heterochromatin mark H3K9me3 at the centromere. However, inducing transcription by an alternative method, namely CENP28/Eaf-mediated elevation of H4K12ac could not rescue the defects associated with loss of H3K4me2, while increasing H3K9ac levels and transcript levels by targeting p65 did rescue the defects. The authors conclude that transcription alone is not sufficient for centromere propagation and function. They propose that transcription combined with the H3K9ac modification is sufficient for new CENP-A assembly, and is necessary to prevent spreading of heterochromatin/H3K9me3 spreading into the centromere.

Overall, this is a well-conceived and executed study that provides important new information about epigenetic regulation of centromere assembly, maintenance and function. However, I have some concerns about the chromatin fiber experiments, as well as the validity of some of the conclusions. Nevertheless, I heartily recommend publication in Nature Communications once the following issues have been addressed.

We thank the referee for this thoughtful summary of our MS. We have made every effort to respond positively to all suggestions.

My only technical concern is with the chromatin fibers. The images presented show very strong DNA staining which is likely to represent bundles of fibers. Previous publications using this method rely on analyzing fibers that have very little visible DNA staining, to avoid misinterpretations about the chromatin composition of specific genomic regions.

We are aware that our fibers show stronger DNA staining than in some other publications using this methodology. However, it is important to take into account that we are working with mitotic fibers rather than interphase fibers, as is usually done when applying this methodology. Indeed, previous publications from our group showed that centromeric fibers (as judged by the CENP-A signals) derived from mitotic cells measured up to 3 µm in length and those derived from interphase cells showed a three to four-fold higher level of stretching that could reach up to 11 µm (PMID: 20483991). We measured the length of the mitotic fibers obtained in our experiments, and we observed a distribution between 0.8 to 5 µm (most of them 2-3 µm), compared to interphase fibers being 1 to 12 µm (most of them 5-8 µm). Therefore, the level of stretching obtained is what would be expected from mitotic fibers.

In this case the specific concern is that conclusions about RNApol2 enrichment at centromeres could arise from the bundling of non-centromeric fibers that contain pol2 with centromeric fibers; in other words, there may be no pol2 on the centromeric chromatin, and results related to the presence of less pol2 after H3K4me2 removal are also called into question. This could also explain why many, but not all 'centromeric' fibers contain pol2. I suggest reanalyzing this data by first screening for tetO signals, then removing fibers that have strong DNA staining from the analysis.

We agree with the referee that the analysis of DNA fibers does require some assumptions, since by the nature of the technique, we cannot know exactly how many strands are present in the fibers. In a separate paper, we have shown by correlative light and electron microscopy that fibers produced under our conditions to appear to be single filaments of 12.6 nm in diameter. But in looking at fibers using fluorescent markers, we cannot know how many DNA molecules are there.

To address the possibility of our fibers containing multiple centromeres, we quantified the levels of ACA signals in mitotic and interphase centromeric fibers. We would predict that

bundles of centromere fibers would have 2x, 3x, etc the amount of ACA staining. In fact, this analysis revealed relatively little variability in ACA fluorescent signal intensities for both interphase and mitotic fibers. This suggests that we are analyzing individual centromere fibers rather than bundles of fibers.

Although we can argue that it is unlikely that multiple centromeres are present, it is much more difficult (probably impossible) to prove that fibers to not contain additional non-centromeric DNA. To do the best that we can on that front, we have followed the suggestion of the Referee.

But, firstly we argue based on our present data that to see an overlap in CENP-C and RNAPII staining as we have for the fiber shown in Figure S2J would require an extraordinary coincidence if this was in fact due to staining from superimposed centromeric and non-centromeric fibers. However, we admit that is only an argument (possibly compelling), and not new data.

We therefore reanalyzed the HAC fibers (as identified by the tetR-EYFP signals) as the reviewer suggested. In order to ensure that we are analyzing fibers that are sufficiently stretched, we made use of the previous quantifications of mitotic fiber lengths and set a threshold of 2 µm (the average length of mitotic fibers) for this re-analysis. HAC fibers with centromere signals shorter than 2 µm were discarded from the analysis. The number of HAC fibers analyzed was increased with fibers captured from a new replicate experiment stained with the same antibodies and tetR-EYFP. The results after re-analysis showed 57% of HAC centromeres with RNAP II-S2ph signal (17 out of 30 HAC fibers). This result is very close to the previously observed 60% of RNAP II-S2ph colocalization.

Very importantly, the results are consistent with those obtained from immunostaining metaphase chromosome spreads, where we observed approximately 50% of metaphases with RNAP II-S2ph staining at centromeres. Therefore the 50% efficiency refers to "intact" centromeres as well as to fibers. Indeed, this is consistent with the results obtained by others staining for RNAP II-S2ph at centromeres of mitotic chromosomes (PMID: 22308327). Furthermore, a personal communication from Dr. Patrick Heun confirmed that these results are also consistent with the frequency at which they observe RNAP-II on mitotic centromeres in Drosophila using GFP-tagged RNAPII subunits.

In addressing this comment, we made numerous attempts to draw further fibers, and have included some of those new images in the revised MS. Those fibers also had detectible DNA, and some were thinner than the ones in our original MS. Using the methods available in our lab, we do not appear to be able to draw thinner fibers while preserving the centromere proteins. We also performed an additional experiment to analyze the frequency of co-localization of RNAP II-S2ph and ACA signals in endogenous centromeres. Results were consistent with the rest of experiments performed so far (IF on unfixed metaphase chromosomes and on HAC fibers): we observed RNAP II-S2ph at centromeres in 45% of fibers analyzed (about half of the centromeres).

New experiments and the new quantitative analysis to address this comment have been included in the Results section (lines 149-155 and 164-167) and we have included a new figure showing more stretched HAC centromere fibers in Figure 2E. We moved the previous pictures of representative HAC fibers to Figure S2J. Other new figures are included in Figures S2D and S2E.

Given the consistency of the data obtained by multiple independent experiments (IF on unfixed metaphase chromosomes, IF on mitotic fibers, IF on HAC fibers) and the new results and quantifications added during this revision, we believe that approximately 50% of metaphases contain RNAP-IIS2ph at centromeres and that it is highly unlikely that bundling of fibers or any other technical artifacts caused the results observed in here.

A related but more minor issue is whether or not these are truly mitotic fibers (see comment below), which could be addressed by incorporating staining for phosphorylated H3 ser10, a mitotic marker that should be retained on the fibers.

Thanks for this helpful suggestion. Although we performed our analysis after adding colcemid and recovering cells by mitotic shake-off, we agree with the reviewer that it was important to prove that these were in fact mitotic fibers. We therefore followed the reviewer's suggestion and performed an experiment preparing fibers from mitotic shake-off or from unsynchronized cultures (as a negative control) and stained them for the mitotic marker H3S10ph. Our results showed a clear difference between shake-off fibers and those obtained from unsynchronized cultures: 95% of the fibers after shake-off were positive for H3S10ph whereas none of the fibers derived from unsynchronized cells showed H3S10ph staining. These results clearly showed that the fibers derived from mitotic shake-off after incubating cells with colcemid were indeed mitotic fibers. These new data were added in the Results section (Lines 148-149) and new panels were added in Figure S2B and S2C.

Examples of conclusions that go beyond the evidence presented:

page 9 bottom: "These data, together with previous results from our group 17 strongly suggest that the CENP-A chaperone HJURP requires H3K4me2 to load new CENP-A molecules at centromeres."
I do not question the result that H3K4me2 is important for new CENP-A assembly, but although HJURP is a key regulator of assembly, the data does not address if HJURP interacts directly with this mark. H3K4me2 could be more directly involved with other proteins or other parts of the assembly pathway. I would change it to '...**suggest that new CENP-A assembly requires H3K4me2**.", or include text that describes the logic more directly.

We agree, and the sentence has been changed as suggested by the reviewer (Lines 198-199).

In the discussion and elsewhere in the manuscript a role for mitotic transcription is emphasized, but it is unclear how the authors are able to differentiate the functional impact of mitotic versus interphase transcription, both of which are claimed to occur at centromeres. Although it is appealing to imagine that the relevant transcription occurs in mitosis, just prior to the onset of new CENP-A assembly, given the evidence presented here and elsewhere it seems equally likely that interphase transcription (at least G2 and S) could be responsible for setting up 'assembly-competent' centromeric chromatin, even if assembly doesn't occur until late mitosis/G1.

The referee is correct. A recent publication from Hongtao Yu's lab (PMID: 26190260) suggested that mitotic centromeric transcription might be differentially regulated and possibly more important in centromere maintenance than interphase transcription. Technically, we cannot detect RNA pol II specifically on the centromeres in interphase cells since it is effectively ubiquitous throughout cell nuclei. We therefore decided to follow up on the centromere transcription during mitosis for our microscopy analysis. In addition, we performed RT-PCR analysis of centromeric transcripts extracted from asynchronous cultures (mostly containing cells in interphase) and from cells blocked in mitosis followed by shake-off. Results showed similar/consistent results in all these experiments. Thus, although we are disrupting centromere chromatin status across the cell cycle, the levels of transcription in mitosis were representative of what we had seen in asynchronous cultures.

Importantly, in our study, we do not claim that mitotic or interphase transcription is more important for CENP-A assembly. Our studies cannot determine this. The main reason for showing both bulk and mitotic transcripts was primarily to confirm that transcripts from the HAC centromere are present in mitosis, and that these transcripts respond to manipulation of H3K4me2 levels similar to transcripts for the log phase population.

minor issues:
p 19: "Importantly, all effects observed on CENP-A levels are corroborated by the effects observed on CENP-C in independent experiments."
This statement is not consistent with the following from p 11, reporting results from tethering CENP28/Eaf and observing increases in CENP-A.
"Interestingly, no corresponding increase was observed in the levels of CENP-C at the alphoidtetO HAC centromere (Figure

S5F, G)."

We thank the referee for pointing this out. The sentence was changed for “whenever we observed decreased levels of CENP-A, these were accompanied by decreased levels of CENP-C in independent experiments” (Lines 416-417).

p 15 top: Although it is clear that p65 tethering results in increased H3K9ac but not H4K12ac, I am confused as to why H3K9ac levels were not increased after tethering of CENP28/Eaf, which also increases the levels of centromeric transcripts? I understand that CENP28/Eaf is a H4K12-specific HAT, but would have expected H3K9ac to increase in response to transcription. What am I missing here?

We were also surprised when we saw no changes on the H3K9ac levels after tethering CENP-28/Eaf6 due to the transcriptional activation, but these experiments have been reproduced multiple times (also during our revisions), so the observation is clearly robust. Although H3K9ac is a typical mark of active transcription, it has recently been shown that H4K12ac can regulate the basal transcription at telomeric heterochromatin regions (PMID: 21249184) and is specifically enriched at the promoters of some specific genes, such as estrogen receptor-alpha (PMID: 25788266). We postulate that our artificial induction of H4K12ac at the HAC centromere might represent another example of this phenomenon. Since the purpose of this MS is not to probe the relative roles of H4K12ac and H3K9ac in transcriptional regulation we would prefer to avoid a speculative discussion of this point in the MS.

p 18: "It has recently been reported that H3K4me2 defines transcription factor binding regions that overlap with promoters or enhancers 49 ."
Although this is true, it is important to note that H3K4me2 is highly enriched over transcribing gene bodies, not just promoters or enhancers, and cannot by itself be used to identify a role for promoters or enhancers, as implied here.

The reviewer is correct and we were not trying to make inferences about promoters or enhancers, both of which would be extremely difficult to map in the repetitive alphoid^{tetO} array. Although we say that LSD2 removes H3K4me2 present at the gene bodies in the introduction, we agree with the referee that it is not clear enough in the discussion when we state that this mark defines transcription factor binding sites. As it is not important for the discussion of our data, we removed this statement from the MS.

Reviewer #2 (Remarks to the Author):

Recent studies have pointed to the importance of active α-satellite RNA transcription and its associated active chromatin marks in directing CENPA deposition by HJURP at the centromere. However, it is yet not fully understood how these epigenetic marks facilitate centromere/CENPA chromatin assembly.

Earnshaw and colleagues have previously demonstrated that H3K4me2 and H3K36me3 are required for maintenance of centromere chromatin. They showed that targeting lysine-specific demethylase 1 (LSD1) led to depleted H3K4me2, resulting in reduced transcription of α-satellite DNA and ability of HJURP/CENPA targeting. In this study, they set to investigate the possible different roles of RNA transcription and active chromatin marks in directing centromere chromatin assembly. They tethered another KMT i.e. LSD2 to the alphoid satellite array on human artificial chromosome (HAC) and found loss of H3K4me2, transcription of α-satellite DNA and HJURP/CENPA targeting. They also investigated the impact of targeting H3K9 acetylation and H4K12 acetylation, in combination with the targeting of LSD2 to the alphoid array on HAC. The 'in situ epistatis' analysis is a good system as it allows the interrogation of how different histone modifications may interact to determine centromere chromatin identity, and pointed by the authors, allows them to uncouple transcription from specific histone modifications. Based on their findings, they conclude that " a balance of mitotic transcription, epigenetic modifications and chromatin remodelling in centrochromatin act as a barrier to prevent heterochromatin spreading and kinetochore inactivation in human centromeres".

We thank the referee for this accurate and favorable description of our results.

In my opinion, the conclusions drawn from the current study are not entirely novel, and are not fully supported by their data. Their previous studies had shown that tethering of the repressive heterochromatin factors caused loss of HAC centromeric transcription and inactivation of its centromere (Cardinale et al., 2009, Nakano et al., 2008, Bergmann et al., 2011, Ohzeki et

al., 2012). The concept that the presence of active chromatin marks is required to maintain centromere transcription, CENPA chromatin assembly is not entirely novel.
The H3K9Ac/H3K9Me antagonistic balance for CENP-A maintenance is not new, in fact the authors had covered it their previous studies (Ozheki et al 2012 and Bergman et al 2012 etc). This detracts from the novelty of the study.

We agree in part with the referee, but would argue that while the present MS builds on earlier work, it goes far beyond that work by developing a system in which we can dissect the relative contributions and roles of histone modifications and transcription.

Despite this, I agree with the authors the importance of investigating the potential different functions of histone modifications (H3K4me2) and RNA transcription in directing centromere chromatin assembly, however, I am not convinced by their data that transcription is not important for CENPA chromatin assembly.

We agree 100% with the referee that transcription and probably the transcripts themselves play a key role or roles in CENP-A chromatin assembly. This comment was actually very helpful to us as it made it clear that we had not explained our arguments sufficiently clearly.

We would like to emphasize that our results do not suggest that transcription is not important on centromere chromatin assembly. Our new experimental approach allowed us to uncouple transcription from epigenetic marks at centrochromatin and therefore to study their separate contribution on centrochromatin assembly. Indeed, our data showed that transcription might have a dual role firstly by stabilizing the centromere chromatin and secondly by introducing H3 acetylation to prevent heterochromatin spreading.

We believe that our data are in agreement with previous observations on the importance of transcription on centrochromatin assembly and stabilization and that our new approach has allowed us to further refine its roles in the complex CENP-A chromatin pathway. We have revised the revised manuscript to make this point clearer throughout the discussion of our results.

As a whole I think this paper have a great potential and the authors have a great system work with, however, further experiments are needed to support their conclusion and there are major areas that need to be addressed:

We thank the referee for this clear assessment and have worked extremely hard and carefully to make both experimental and writing changes, trying to respond in a positive way to every comment made by the referee. We hope that in this revision we have successfully addressed any concerns and problems.

1) The authors showed that expression of p65 which increases H3K9Ac (even in the LSD co-expressing cells, thus having low H3K4me2) resulted in an increase of transcription besides the increase in CENP-A loading. So it is not just specific histone modifications, but also the RNA transcription (Fig 6) that is required for CENPA chromatin assembly.

Targeting of p65 increases H3K9Ac, thus likely also directly prevents the formation of H3K9me3 chromatin. This also agrees with the idea that "...H3K9As directly/indirectly antagonises heterochromatin spreading into centrochromatin" (line 419- 420, p17). Fang et al 2010 showed that LSD2 can direct H3K9 methylation by G9a KMT.

This is why we were careful to make a catalytically dead form of LSD2 to use in important control experiments. It seems clear from our results with the catalytically dead mutant for LSD2 that our chimeric fusion proteins are not acting by attracting G9a KMT, at least not at levels that destabilize the centromere. In particular, tethering a catalytically dead LSD2 mutant to the HAC did not change the levels of H3K4me2 and other epigenetic marks associated with transcription (Figure 1E). Moreover, it did not destabilize the kinetochore after a long period of tethering, as CENP-A and CENP-C levels were maintained for up to 10 days after tethering it to the HAC centromere (Figure 3A, D, E).

The inability of the increase in H4K12Ac and active transcription in CENP28 expressing cells to direct CENPA loading or centromere chromatin assembly may be because H3K9me (due to LSD2 targeting) is enriched at HAC centromere in both LSD2, LSD2/cenp28 expressing cells. Whereas, in LSD2/p65 expressing cells, the increase in H3K9Ac by p65 can antagonise the formation of H3K9me3 chromatin. Furthermore, p65 targeting also results in the increase in transcription at

HAC centromere. The conclusion that p65 induced H3K9Ac, but not cenp28 induced H4K12Ac is sufficient to bypass the requirement of H3K4me2 is not entirely correct. It is most likely that both H3K9Ac (and the lack of H3K9me3) and transcription (promoted by H3K4me2 or H4K12Ac) are required for CENPA chromatin assembly.

This is a good point. We agree with the reviewer that the effects are complicated, and we have tried to explain this more clearly in the revised MS. We absolutely agree that transcription (and probably the transcripts) is important and that the interplay between histone modifications is also important. Indeed we agree with the referee that H3K4me2 ***and*** H3K9ac are probably both important for centromere maintenance - H3K4me2 because it somehow promotes or reinforces centromeric transcription, and H3K9ac because it keeps H3K9me3 from spreading.

To further examine the latter, we were inspired by the reviewer to test the effects of tethering LSD2+CENP-28 and LSD2+p65 on H3K9me3 spreading into centrochromatin. We performed immunofluorescence on unfixed metaphase chromosomes, staining for H3K9me3 and CENP-A after expressing LSD2+CENP-28 and LSD2+p65 as we did previously after expressing LSD2. The results were entirely consistent with our previous data showing that the destabilization of centrochromatin when expressing LSD2+CENP-28, was accompanied by spreading of heterochromatin on the HAC centromere (similar to what we observed tethering LSD2 to the HAC). In contrast, expressing LSD2+p65, restored a normal distribution of H3K9me3 at the HAC centromere (similar to what we observed in the control cells maintained with doxycycline – no LSD2 tethering-).

Thus, in agreement with the reviewer's suggestion, these new results suggest that transcription has a role in regulating the balance of histone modifications such as H3K9ac to prevent heterochromatin spreading into CENP-A chromatin.

We suggest that this makes it all the more interesting that transcription coupled with H4K12ac without H3K9ac was not able to antagonize the formation of H3K9me3. This serves to further emphasize the importance of H3K9ac introduced by transcription. These new results were introduced in the manuscript at the Results section (lines 377-385) and the discussion was amended addressing this point more clearly. Moreover new panels were included in Figure 8C and D.

2) Targeting p65 increased RNA transcription at the HAC centromere in LSD2 tarted cells, but not in CENPA loading. Would the authors look into the presence of H4K20me1 in LSD2, LSD/p65, LSD2/CENP28 expressing cells. They have shown that H4K20me2 is essential in CENP-A chromatin assembly (Hori et al 2014 Dev Cell). Does targeting of p65 affects H4K20me1 formation, thus resulting in a reduced ability of CENPA chromatin assembly even with restored RNA transcription activity? Also what about H3K36me3 which is a slo found to be associated with centromere transcription by a previous study (Bergman et al 2012).

Targeting p65 increased transcription in LSD2 targeted cells and also the CENP-A loading. Probably the reviewer meant targeting CENP-28?

Following this suggestion of the referee we have looked at the fate of both H3K36me2 and H4K20me1 in our system. Results with the former were as expected, but the experiments with H4K20me1 were less informative.

H3K36me2 is a transcription-associated mark that is present at centromeres (PMID: 21157429). We performed ChIP experiments, and consistent with the effects on transcription, we observed a moderate decrease in the levels of H3K36me2 after tethering LSD2 to the HAC compared with the control cells expressing tetR-mCherry. As expected, H3K36me2 recovered to normal levels after expressing LSD2+p65. H3K36me2 did not recover after expressing LSD2+CENP-28. These results were added to the Results section (lines 357-360) and we included an entire new figure as Supplementary Figure 7.

The situation with H4K20me1 was more complex, as this mark was present at much lower levels on centromeres than H3K4me2 or H3K36me2. It was also lower on the HAC than on the endogenous chromosome 21 used as a control. It should therefore be borne in mind

that all studies of this mark had a very minimal signal-to-noise ratio, and were only borderline reliable. Results of our new experiments showed a mild reduction of H4K20me1 levels after tethering LSD2 to the HAC compared with the levels of the control cells expressing tetR-mCherry. Levels of H4K20me1 remained low after expressing both LSD2+CENP-28 and also LSD2+p65. Thus H4K20me1 levels on the HAC do not appear to correlate strongly with CENP-A assembly and maintenance. In a previous collaborative study, we showed that the removal of H4K20me1 from centromeres did not affect the levels of CENP-A or CENP-C, but did affect the levels of CENP-H (PDMI: 24960696). At that time, we postulated that H4K20me1 might be important for kinetochore stabilization rather than a role on CENP-A loading.

Our new results, although difficult to interpret due to the low levels of H4K20me1 at centromeres, are consistent with these data and suggest that this mark does not have a direct effect on the CENP-A assembly pathway. It is possible that it might play a role in the maintenance of a mature kinetochore upstream of CENP-A loading or in promoting transcription, which is bypassed by both CENP-28 and p65 tethering.

We have briefly mentioned the H3K36me2 result in the Results section (lines 357-360) and included an entire new figure as Supplementary Figure 7. However, in the interest of saving space and minimizing unnecessary complications, we therefore request that we be allowed to omit the data for H4K20me1, since it did not add any further insights to our story.

3) Re: pg 9, line 222-224, Could authors show changes in HJURP levels in the LSD2, LSD2/p65, LSD2/cenp-28 targeted cells? Their previous studies showed that H3K4me2 being important for HJURP recruitment (Bergmann et al 2011). Since CENP-A levels are also increased in LSD2/p65 (so high H3K9AC and low H3K4me2) expressing cells. This would clear up the uncertainty if it's just CENP-A loading and/or stabilisation that is affected. This is important as CENP-A chromatin propagation is a multistep complex pathway: priming of chromatin, removal of placeholders, recruitment of CENP-A loaders, loading of CENP-A, and stabilisation (i.e RSF and more importantly HATs required for Mis18, but insufficient for HJURP etc see Ohzeki 2012). Many of these other steps were not investigated in the current study.

This was another helpful point. As the reviewer suggested, we tried to clear up the uncertainty of whether it is the CENP-A loading or its stabilization what is affected after tethering LSD2, LSD2+CENP-28 and LSD2+p65. We first tested two different HJURP antibodies with multiple different fixations (methanol, formaldehyde), but unfortunately we did not manage to obtain a good staining with any of them. Moreover, we could not use the construct used in our previous study as it is fused with RFP and it interferes with the red tags of our *in situ* epistasis assays.

Therefore, we decided to address this interesting question using a more powerful functional approach rather than simply antibody staining. We employed pulse-chase experiments with two different protocols adapted to study the loading of newly synthesized CENP-A molecules and the stability of the CENP-A molecules at the HAC centromere after tethering LSD2, LSD2+CENP-28 and LSD2+p65. This enabled us to look directly at the key point raised by the referee.

Since p65 is fused with the SNAP tag, we used Halo-tagged CENP-A for these analyses. Halo substrates are available which are compatible with the labeling of the SNAP-tag with TMR-Star. Results on the loading of newly synthesized CENP-A molecules were very clear, and consistent with our initial results using CENP-A-SNAP. We observed decreased levels of new Halo-CENP-A loading after tethering LSD2 to the HAC centromere compared with the control. Tethering LSD2+p65 recovered the loading of new Halo-CENP-A molecules, but they were clearly NOT recovered after tethering LSD2+CENP-28. This suggested that depletion of H3K4me2 decreases the loading of new CENP-A molecules at centromeres and that this loading could be restored by transcription coupled with H3K9ac but not with transcription coupled with H4K12ac. This is completely consistent with the “defensive” role for H3K9ac described in our response above.

Regarding the stability of Halo-CENP-A at the HAC centromere, we observed decreased levels of Halo-CENP-A at centromeres after expressing LSD2. Remarkably, transcription coupled with ***either*** H3K9ac or H4K12ac bypassed the requirement for H3K4me2. These results suggested that the transcription process or the transcripts (or both) have a role in the stabilization of CENP-A molecules at centromeres. These data are in agreement with other authors suggesting that centromeric ncRNAs stabilize CENP-A (PMID: 25117489 and 25365994) and CENP-C molecules (PMID: 25954010 and 20140237) at centromeres. The new results were added to the Results section (lines 322-336) and Discussion (lines 457-463). New figures with representative microscopy images and quantification data of these experiments were included in Figure 7C,D and E.

4) Was level of CENPC looked at in cells expressing LSD2/cenp28 (Fig 5)?

Yes, the levels of CENP-C after expressing LSD2/CENP-28 were analyzed and are included in Figure S5F,G.

5) A significant loss of transcripts is noted after 2 days of LSD2 expression, but RNA Pol2 and H3K4me2 levels only became significantly reduced after more than 4 days? Similar observation was made with CENP-A and C levels. Could this indicate that transcription and H3k4me2 are somewhat uncoupled and serve separate function. Perhaps transcription may be responsible for remodelling/priming the chromatin initially and also the reduction of transcripts may have a role in remodelling the chromatin as well (as suggested previously by Quenet 2014 eLife).

This is an important question. However, the levels of H3K4me2 are significantly reduced after tethering LSD2 for 24h and the levels of centromeric transcripts after LSD2 tethering are significantly lower only after 48 hours. This suggests that that transcription falls ***after*** H3K4me2 removal, but before the levels of CENP-A fall.

The levels of RNAP II drop in immunostaining experiments only after 4 days of LSD2 tethering (similar to the kinetics of CENP-A loss). This difference in timing may be due to the presence of stalled RNAP II on the centromeres. Other authors have recently showed that centromeres can contain stalled RNAP II that triggers chromatin-remodeling activities to promote CENP-A deposition (PMID: 25738810). Together all of these observations are consistent with the hypothesis that the subset of Pol II that is actively transcribing decreases but the effect on overall RNA Pol II levels is observed only later, as it takes more time to visualize this decrease in the presence of the stalled RNAP II at the centromeres. We added a sentence to address this point in the Results section (lines 142-143).

6) Fig 8 showed a greater increase of H3.3 loading in LSD2/cenp28 expressing cells. This increase in H3.3 loading also correlates with the higher RNA transcription in these cells (nearly 4 fold increase), when compared to that in LSD2/p65 expressing cells. Is it possible that the increase in H4k12Ac promotes a RNA pol2 activity that is too high, hence, affecting the loading/stabilisation of CENPA. Only a low level of transcription at the centromeres, a high level of RNA Pol2 may not be compatible with the maintenance of CENPA stability. Could authors consider modifying RNA pol2 targeting or activity to the HAC centromere using their system, without affecting the presence of H3K9Ac and/or H3K4me2? This would be useful to determine if RNA transcription is essential for CENPA loading and in other steps (priming, stabilisation, RSF positiioning etc.

This is a very good point and we developed a novel strategy to address it. We did not feel it wise to embark on a whole new series of targeting experiments for RNAPII, and we do not feel that this MS is the place to try and examine the roles of H4K12ac and H3K9ac on RNAPII transcription.

So instead we focused on the primary concern of the referee, which we would rephrase to say – how do we exclude that tethering CENP-28 to the HAC in some way interferes with CENP-A incorporation? – for example by stimulating high levels of transcription.

We had not originally been concerned by this possibility, because we had previously shown that in the presence of H3K4me2, centromeres can tolerate a 10x increase in transcription – significantly greater than the 4-fold increase caused by tethering CENP-28. Indeed centromeres were only destabilized after increasing transcription by approximately

150-fold (PMID: 22331359). Furthermore, we have shown here that tethering CENP-28 alone (in the presence of H3K4me2) actually increased the levels of CENP-A in the HAC centromere (Figure 5E and F). Thus CENP-28 is not *per se* harmful for CENP-A incorporation.

However, since CENP-28 increased transcription levels slightly more than p65, we agreed with the reviewer that this issue should be addressed. Since we could not modify RNAPII targeting or activity on the HAC, we decided to address this question by further expanding the *in situ* epistasis assay - by tethering ***<u>three</u>*** competing activities to the same alpha-satellite tetO-array. Remarkably, this experiment worked perfectly. We tethered LSD2, LSD2+CENP28 or LSD2+CENP28+p65 in parallel experiments and analyzed the levels of CENP-A at the HAC centromere. As expected, tethering LSD2, LSD2+CENP28 caused CENP-A levels to drop on the HAC. Remarkably, levels of CENP-A at the HAC centromere were completely restored after tethering LSD2+CENP28+p65. These results strongly suggest that the destabilization of the centromere after tethering LSD2+CENP-28 is not due to an excess of centromeric transcription.

This experiment further demonstrates the versatility of our *in situ* epistasis approach. If it is informative to tether three (and possibly several more) activities to the HAC, it might be possible to use this system in the future to reconstitute even more complex pathways involved in centromere assembly or stability. These data have been incorporated in the Results section (Lines 342-352) and new panels were included in Figure 8A,B.

Reviewers' Comments:

Reviewer #1 (Remarks to the Author):

The authors have done an amazing job of addressing all of my concerns. For example, they went to great lengths to address concerns about the mitotic fiber analyses, and provide new convincing evidence that completely addresses the issue. Further, concerns about inappropriate conclusions have been addressed with text revisions. I heartily recommend acceptance for publication in Nature Communications.

Reviewer #2 (Remarks to the Author):

Earnshaw and colleagues have adequately addressed major points raised in the previous review. In the revised manuscript, they have provided supporting evidence that presence of H3K4me2 is required for maintenance of transcription and CENP-A loading at centromeres. Although it remains puzzling why H3K9 acetylation is not increased (despited the restoration of transcription and H4K20 acetylation) following targeting of LSD2/CENP28, their findings provide evidence that H3K9 acetylation is required for preventing H3K9me3 spreading.

It is noted that H3.3 level remains high following LSD2 and LSD2/CENP28 targeting. So, could the increase of H3K9me3 caused by K9 methylation of the placeholder H3.3? Could the authors comment on this?

In addition, could the authors comment (add in the discussion section) how presence of H3k4me2 may facilitate H3k9 acetylation (and prevents H3K9me3 spreading) given transcription activity alone (for example, by Cenp28 targeting) is insufficient to promote H3k9 acetylation? Do they think histone acetyltransferases may be directly recruited and when does this occur during CENPA chromatin assembly? Also, could the authors discuss this in light of their previous finding that targeting of tetR-EYFP-p300 or tetR-EYFP-PCAF, two histone acetyltransferase domains that promote acetylation of H3K9, results in assembly of new CENPA chromatin (Ohzeki et al 2012).

Response to referees:

Reviewer #1 (Remarks to the Author):

The authors have done an amazing job of addressing all of my concerns. For example, they went to great lengths to address concerns about the mitotic fiber analyses, and provide new convincing evidence that completely addresses the issue. Further, concerns about inappropriate conclusions have been addressed with text revisions. I heartily recommend acceptance for publication in Nature Communications.

We thank the referee for his/her thoughtful suggestions during the revision process. They significantly improved our paper.

Reviewer #2 (Remarks to the Author):

Earnshaw and colleagues have adequately addressed major points raised in the previous review. In the revised manuscript, they have provided supporting evidence that presence of H3K4me2 is required for maintenance of transcription and CENP-A loading at centromeres. Although it remains puzzling why H3K9 acetylation is not increased (despited the restoration of transcription and H4K20 acetylation) following targeting of LSD2/CENP28, their findings provide evidence that H3K9 acetylation is required for preventing H3K9me3 spreading.

We also thank this referee for his/her thoughtful suggestions during the revision process.

It is noted that H3.3 level remains high following LSD2 and LSD2/CENP28 targeting. So, could the increase of H3K9me3 caused by K9 methylation of the placeholder H3.3? Could the authors comment on this?

We agree with the referee that this is a potential possibility. Comparison of the two sequences:

H3.3 ARTKQTARK STGGKAPRKQ LATKAARKSA PSTGGVKKPH

H3.1 ARTKQTARK STGGKAPRKQ LATKAARKSA PATGGVKKPH

Does not reveal differences in the immediate environment of K9, so it is possible that antibodies reacting with H3.1K9me3 might also cross-react to H3.3K9me3. However, since we have not tested this possibility explicitly we would prefer not to mention this in the paper.

In addition, could the authors comment (add in the discussion section) how presence of H3k4me2 may facilitate H3k9 acetylation (and prevents H3K9me3 spreading) given transcription activity alone (for example, by Cenp28 targeting) is insufficient to promote H3k9 acetylation? Do they think histone acetyltransferases may be directly recruited and when does this occur during CENPA chromatin assembly? Also, could the authors discuss this in light of their previous finding that targeting of tetR-EYFP-p300 or tetR-EYFP-PCAF, two histone acetyltransferase domains that promote acetylation of H3K9, results in assembly of new CENPA chromatin (Ohzeki et al 2012).

We thank the referee for this suggestion and have modified our Discussion in two places.

In the paragraph starting on line 397 we now say:

"Recent results have revealed that centromeres undergo low levels of RNAP II-mediated transcription during mitosis [36,38]. We confirmed these results for the HAC and further showed that H3K4me2 depletion affects levels of both centromeric mitotic transcripts and centromere-associated RNAP II (Figure 9a). Many transcription factors appear to read the H3K4 methylation

mark: in one analysis, over 90% of transcription factor binding sites were found to map within regions of increased H3K4 methylation [49]. Specifically, Sgf29 binding to H3K4me2/3 has been reported to recruit the SAGA complex and promote histone H3 acetylation [50]. At centromeres this acetylation could be linked with licensing for new CENP-A assembly, as seen with p300 and PCAF acetyltransferase domains were targeted to the alphoid^{tetO} array [39]. In addition, the chromatin remodeller CHD1 also binds H3K4me2 [49] and this could promote RNAP II activity associated with H3 acetylation at centromeres during mitosis. Indeed, CHD1 depletion has been shown to decrease CENP-A incorporation and disrupt centromere function [50]. "

Later on in the paragraph starting on line 424 we have added the following information:

"The linking of centromere stability to multiple chromatin marks and to the process of transcription (or to the transcripts themselves) reveals a complex system for centromere maintenance. Epigenetic marks may maintain centrochromatin stability by recruiting factors such as RSF [54] and/or MgcRacGAP [55]. In other experiments, our laboratories recently found that the chromatin remodelling factor RSF, recruited by acetylation of histone H3 can promote CENP-A incorporation at an ectopic site [56]. "

New references:

50. Bian, C. *et al.* Sgf29 binds histone H3K4me2/3 and is required for SAGA complex recruitment and histone H3 acetylation. *The EMBO journal* **30**, 2829-2842, doi:10.1038/emboj.2011.193 (2011).
